# An RNA pseudoknot is essential for standby-mediated translation of the *tisB* toxin mRNA in *Escherichia coli*

**DOI:** 10.1093/nar/gkaa1139

**Published:** 2020-11-24

**Authors:** Cédric Romilly, Anne Lippegaus, E Gerhart H Wagner

**Affiliations:** Department of Cell and Molecular Biology, Uppsala University, Uppsala S-75124, Sweden; Department of Cell and Molecular Biology, Uppsala University, Uppsala S-75124, Sweden; Department of Cell and Molecular Biology, Uppsala University, Uppsala S-75124, Sweden

## Abstract

In response to DNA damage, *Escherichia coli* cells activate the expression of the toxin gene *tisB* of the toxin–antitoxin system *tisB-istR1*. Of three isoforms, only the processed, highly structured +42 *tisB* mRNA is active. Translation requires a standby site, composed of two essential elements: a single-stranded region located 100 nucleotides upstream of the sequestered RBS, and a structure near the 5′-end of the active mRNA. Here, we propose that this 5′-structure is an RNA pseudoknot which is required for 30S and protein S1-alone binding to the mRNA. Point mutations that prevent formation of this pseudoknot inhibit formation of translation initiation complexes, impair S1 and 30S binding to the mRNA, and render the *tisB* mRNA non-toxic *in vivo*. A set of mutations created in either the left or right arm of stem 2 of the pseudoknot entailed loss of toxicity upon overexpression of the corresponding mRNA variants. Combining the matching right-left arm mutations entirely restored toxicity levels to that of the wild-type, active mRNA. Finally, since many pseudoknots have high affinity for S1, we predicted similar pseudoknots in non-homologous type I toxin–antitoxin systems that exhibit features similar to that of *tisB-IstR1*, suggesting a shared requirement for standby acting at great distances.

## INTRODUCTION

**Figure 1.**
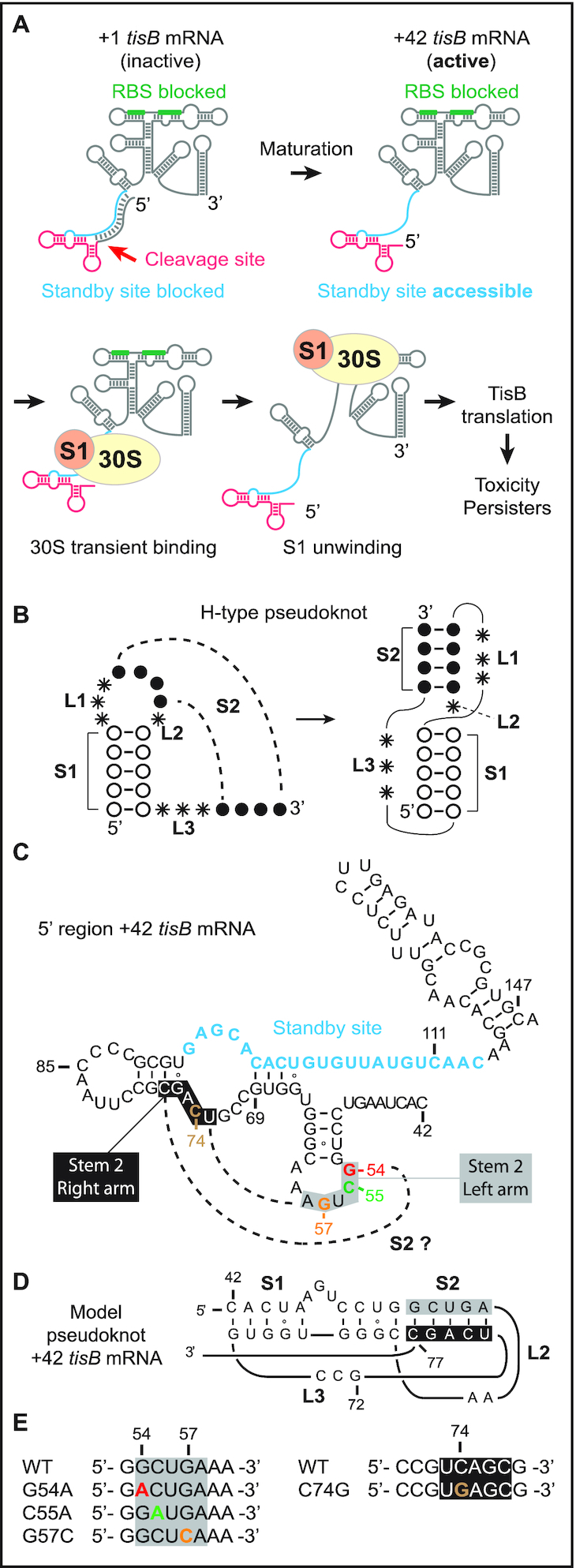
Model of standby-mediated translation of *tisB* mRNA involving a pseudoknot. (**A**) The steps involved in translation of *tisB* mRNA are detailed in the Introduction. The sequestered *tisB* RBS (green) and single-stranded standby site (blue) are indicated. Translation requires transient S1-guided 30S subunit binding to the standby site to promote structure unwinding towards the RBS. The functional standby region contains a single stranded region (blue) and a 5′-end structure (red). (**B**) Schematics of an H-type RNA pseudoknot showing characteristic stem (S1 and S2) and loop (L1, L2 and L3) features. (**C**) Secondary structure of the 5′ end region of +42 *tisB* mRNA with nucleotide sequence information. The unstructured standby site is in blue. Dashed lines indicate base-pairing that would generate a pseudoknot via formation of a second stem, S2 (grey box: left flank of stem; black box: right flank). (**D**) Pseudoknot model schematic for the 5′ end segment of +42 *tisB* mRNA, based on HotKnots software predictions (see Results). General features: S1 = 9 nt, S2 = 5 nt, L1 = 0 nt, L2 = 2 nt and L3 = 3 nt) (**E**) Point mutations used in this work. Color code as in C.

In enterobacteria like *Escherichia coli*, DNA damage induces the SOS stress response genes, one of which encodes the toxin of the type I toxin–antitoxin (TA) locus *tisB-istR1*. During normal growth, *tisB* is repressed by the master regulator LexA (1–3). It encodes a pore-forming toxin that arrests growth through membrane depolarization and promotes the formation of persisters in response to stress (4–7). Persisters are non-growing, yet sometimes metabolically active cells that tolerate antibiotic treatment and counteract host immune responses (8,9). The *tisB* primary transcript, denoted +1 *tisB* mRNA (Figure 1A), is translationally inactive since the *tisB* ribosome binding site (RBS) is structurally sequestered (10,11). Maturation by endonucleolytic cleavage gives rise to the +42 *tisB* mRNA. Despite an identically sequestered RBS, this mRNA is translationally active, and hence confers toxicity to cells when overexpressed (4,10).

The apparent paradox of mRNAs with structurally inaccessible RBS’s was solved conceptually by the ‘ribosome standby’ model (12,13). We showed previously that the +42 *tisB* mRNA, but not the inactive +1 variant, carries a largely unstructured segment far upstream of the *tisB* RBS. The 30S ribosomal subunit must bind this site to subsequently move towards the RBS located ≈100 nt downstream (11,14). We learned that this standby site is composed of two distinct elements: a single-stranded region and a 5′ terminal structure (14). Both are required for standby-mediated translation of *tisB* mRNA. Under non-stress growth conditions, the low, uninduced levels of +42 *tisB* mRNA are inhibited by the constitutively transcribed antitoxin RNA IstR1. IstR1 base-pairs to the single-stranded region near the 5′-end, thus preventing standby and, thereby, TisB translation and toxicity (10,11). We recently showed that a structure near the 5′-end of +42 mRNA was a second essential element for standby. Mutations that disrupted the 5′-most stem-loop, or lacked it altogether, severely impaired ribosome binding to, and translation of, *tisB* mRNA. This paper also showed that ribosomal protein S1 alone binds to both standby elements, is needed to promote 30S subunit binding to this location, and facilitates the unwinding of downstream structure to reach the *tisB* RBS (Figure 1A) (14).

The importance of S1 for standby is intriguing. In *E. coli*, this protein has ubiquitous roles in translation (15–18). It is the largest ribosomal protein, and is not always stably associated with ribosomes (18,19). Through its six OB-fold domains, it binds many RNA elements (20), e.g. single-stranded AU-rich sequences near RBS’s of mRNAs to enhance translation (21–23). Conversely, since S1 also recognizes complex RNA structures, its binding and unwinding activity is sometimes required for translation of structured mRNAs (24–29). Of interest for the question addressed here, S1 avidly binds RNA pseudoknot structures, which involve hairpin apical loops base-pairing to other, often flanking, single-stranded RNA segments. The most prevalent pseudoknot, denoted H-type, displays co-axial stacking of two stem regions (Figure 1B; stems S1 and S2), connected by three loop regions (Figure 1B; L1, L2, and L3) (30–32). These RNA structure elements are often critical for regulation of gene expression in viruses, pro-, and eukaryotes (33,34). Pseudoknots are structurally diverse, differing in topology due to variations in the length of the loops and helices that they are built from (31,32,35). Early evidence of S1/RNA pseudoknot interactions was obtained by SELEX experiments which identified an H-type RNA pseudoknot bound by S1 with nanomolar affinity (Supplementary Figure S1) (36). Subsequently, S1 was shown to bind pseudoknots in tmRNA (37–39), the preQ_1_ riboswitch (40), and the *rpsO* mRNA. In the latter case, binding is essential for unwinding and docking of the mRNA onto the 30S subunit platform (26) (Supplementary Figure S1).

Our recent work revealed that a structure at the 5′-end of +42 *tisB* mRNA is required for S1 binding and standby activity. Given the preference of S1 for RNA pseudoknots, we speculated that such an element might explain how this ribosomal protein anchors the 30S subunit to facilitate high affinity binding to the entire standby site. Here, we present results that support this hypothesis, by demonstrating that point mutations in the apical loop of the 5′-end stem-loop reduce affinity of the mRNA for 30S subunits and purified S1. Loss of binding is associated with impaired 30S initiation complex (30S-IC) formation *in vitro* and alleviated toxicity *in vivo*. A compensatory mutation in a flanking single-stranded loop is suggested to restore the pseudoknot, hereby allowing translation of the inactive mutant mRNA. We corroborated our conclusions by analyzing three additional pairs of double-nucleotide mutations in stem 2 of the predicted pseudoknot. *In vivo* toxicity was abolished in all cases, but restored by the compensatory mutations. Finally, we suggest that toxin mRNAs from non-homologous, but similarly organized, TA systems also carry predicted RNA pseudoknots at equivalent locations.

## MATERIALS AND METHODS

### Bacterial cultures

Strains and plasmids are listed in Supplementary Table S1. Cells were grown aerobically at 37°C in L Broth (5 g/l yeast extract, 10 g/l tryptone,10 g/l NaCl) and, when required, ampicillin was added at 100 μg/ml or kanamycin at 50 μg/ml. Plasmids pBAD, pBAD+1_*tisB* and pBAD+42_*tisB*, as well as pJV974–1, were published previously (4,10). The pBAD plasmid series was used as template for site-directed mutagenesis with primer design based on NEBaseChanger (http://nebasechanger.neb.com/). After PCR amplification with phosphorylated primers, template plasmids were digested with FastDigest *Dpn*I (ThermoFischer Scientific, FD1703) for 30 min at 37°C followed by purification of the PCR products with the GeneJET PCR Purification Kit (ThermoFisher Scientific). Plasmids were re-circularized with T4 DNA ligase (ThermoFischer Scientific, 15224017) and transformed into chemically competent *Escherichia coli* Top10 cells (C404003, Invitrogen). The mutated pBAD plasmids were isolated with the GeneJET Plasmid Miniprep Kit (ThermoFisher Scientific).

### Toxicity assays

Overnight bacterial cultures derived from single colonies were diluted 1:100 in fresh LB medium and grown in either 100 ml flasks or black 96-well plates with clear flat bottom (Costar^®^) at 37°C. Optical density (600 nm) was measured at 5 min intervals in a plate reader (Tecan infinite pro). At OD_600_ = 0.5, either arabinose or glucose (0.2% or 0.025% final concentration) was added to induce or prevent induction of expression from the pBAD plasmids (Supplementary Figure S1). OD values were corrected for media-only background. For spotting assays, an aliquot of a 30 min induced culture with either glucose or arabinose was withdrawn, and 10-fold serially diluted in fresh LB media. 5 μl aliquots were pipetted on fresh LA-Amp plates, followed by o.n. incubation at 37°C. Images were captured using a Chemidoc (Biorad, image lab 4.0 software).

### Northern blot analysis

Aliquots from bacterial cultures with either arabinose or glucose were withdrawn and mixed with 0.25 volume of 95% ethanol, 5% phenol, and frozen in liquid nitrogen. After thawing on ice, cells were pelleted by centrifugation (15 min at 4000 g) and RNA extracted by hot-phenol (41). 10 μg of total RNA was mixed with 2 × RNA loading buffer (95% (v/v) formamide, 0.025% (w/v) bromophenol blue, 0.025% (w/v) xylene cyanol), denatured for 2 min at 90°C, and separated on a 6% sequencing gel. After electrophoresis, RNAs were transferred to an Amersham HybondTM-N+ membrane (GE Healthcare) by electro-blotting, and UV-crosslinked. 5′-end-labeled oligodeoxyribonucleotide probes were used for detection of *tisB* mRNAs (ced267) and the 5S rRNA (5S-long). Pre-hybridization and hybridization of the membrane was carried out in Church and Gilbert hybridization buffer (Church & Gilbert, 1984) at 50°C. Signals were detected using a PhosphorImager screen and a PMI scanner™ (Biorad).

### RNA preparation

DNA templates with T7 promoter sequences were generated by PCR with hot start Phusion (primers in Supplementary Table S2). Transcription of RNAs was done with the Megascript kit (Life technologies, #AM1330) according to the manufacturer's instructions. The encoded hammerhead ribozyme (HH-ribozyme) sequence was completely removed with 10 cycles of 3 min at 60°C followed by 3 min at 25°C in a PCR block, after Turbo DNase treatment. The general template design for specific cleavage using the HH-ribozyme is shown in Supplementary Figure S2. RNAs were purified from 6% sequencing gels, and RNA bands detected by UV-shadowing. RNA was passively eluted in elution buffer (300 mM Na-acetate, 0.1% SDS, 1 mM EDTA) o.n. at 4°C, followed by phenol extraction, ethanol precipitation, and drying. Dried RNA pellets were dissolved in RNAse-free water, concentration obtained by Nanodrop, and quality evaluated by SybrGold staining after denaturing PAGE.

### Fluorescein (FAM) labeling of mRNAs

The strategy used for FAM-labeling of RNA is described in (14). Briefly, 5′OH +106 RNA generated by hammerhead ribozyme cleavage (see above), was splint-ligated to FAM-RNA adaptors to reconstitute the 5′FAM +42 *tisB* mRNA (idtDNA) following the strategy described in (42,43) with modifications listed hereafter. 300 pmol of 5′OH +106 RNA was incubated with 25 U of T4 PNK (Thermo Scientific™, EK0031) and 0.5 mM ATP for 1 h at 37°C to mono-phosphorylate the 5′-end of the mRNA. ATP and ADP were removed on Illustra MicroSpin G50 columns (GE Healthcare, 27-5330-01), followed by phenol extraction and precipitation. Next, 300 pmol of phosphorylated RNA, 450 pmol of splint-DNA oligo, and 600 pmol of FAM-adaptor were mixed in a 25 μl volume. For RNA/DNA heteroduplex formation, the reaction mix was denatured for 2 min at 95°C, followed by slow cooling to r.t. The reaction mix was supplemented (total volume 50 μl) with 1x T4 RNA ligase buffer (Thermo Scientific™, EK0013), water, 10 U of SUPERase•In™ RNase Inhibitor (Invitrogen™, AM2696), 8% PEG4000, and 360 Weiss Units of T4 DNA ligase (Thermo Scientific™, EK0013). Ligation was carried out o/n at 30°C, followed by phenol extraction and precipitation. FAM-ligated RNAs were purified on 6% sequencing gels. As a control, incorporation of fluorescein was confirmed using a Chemidoc (Biorad, image lab 4.0 software, fluorescein settings). Bands corresponding to the desired RNA sizes were cut out, and RNA eluted o.n. at 4°C as described above.

### Ribosome and S1 purification

30S ribosomes and ribosomal protein S1 were purified as in (14).

### Toeprint assays

Toeprint assays were done according to (14). All steps were at 37°C unless otherwise specified. Before use, each mRNA together with dNTPs (0.5 mM final concentration) and radiolabeled ced70 primer (150 000 cpm) were denatured for 1 min at 90°C, followed by 1 min on ice. RNAs were refolded for 10 min in toeprint reaction buffer (10 mM Tris–HCl pH 7.6, 100 mM K-acetate, 10 mM Mg-acetate, 1 mM DTT). Pre-activated 30S (100 nM final concentration; 15 min at 37°C in 1× RT-buffer) were added for 10 min, followed by addition of tRNA^fMet^ (300 nM). 30S-IC formation was allowed for 20 min. Primer extension was carried out by addition of 50 U of SSIV RT (Invitrogen). After phenol/ chloroform/ isoamyl alcohol extraction (25/24/1), template RNAs were hydrolyzed by KOH treatment (300 mM) for 3 min at 90°C, followed by 1 h at 37°C. KOH was neutralized with acetic acid (600 mM), and the cDNA precipitated with Na-acetate and ethanol. After centrifugation and washes with 70% ethanol, cDNA was dissolved in 1 vol of water and 1 vol of 2× RNA loading dye (R0641, Thermo Fisher) and separated on an 8% sequencing gel. Signals were detected using a PhosphorImager screen and a PMI scanner™ (Biorad).

### 30S and S1 binding assays

30S subunit and S1 binding to the +42 *tisB* mRNA was assessed by measuring fluorescent anisotropy change with a 5′end FAM-labeled +42 mRNA. 10 nM of labeled mRNA was denatured and refolded as described above at 37°C for 15 min. 30S subunits or purified S1 (pre-incubated in toeprint reaction buffer at 37°C) were added to the reaction mix with or without competitor RNA (50-fold molar excess of unlabeled mRNA only for 30S binding assays, 50-fold and 10-fold molar excess of unlabeled mRNA, respectively, for S1 binding assays). Complex formation was allowed for 20 min at 37°C (final assay volume 50 μl). Three 15 μl aliquots were loaded on 384-well plates. Fluorescein was excited with polarized light at 485 nm and polarized parallel and orthogonal emission at 535 nm was recorded using a Tecan SPARK 10M. Anisotropy was calculated with the following equation:

$$\begin{equation*}Anisotropy = \ \frac{{\parallel - \bot }}{{\parallel \ + \ 2 \times \bot }}\end{equation*}$$

where (∥) is parallel and (⊥) is orthogonal polarized emission, respectively. All experiments were done in triplicate.

### SHAPE structural probing

Reactions were done in a final volume of 15 μl. 2 pmol of RNA was denatured for 1 min at 90°C followed by 2 min incubation on ice. RNA was refolded for 5 min at 37°C in MKM buffer (HEPES 50 mM pH 8, Mg-acetate 5 mM, K-acetate 100 mM). Probing was carried out for 70 s at 37°C in presence of 1.5 μl of DMSO only or 1.5 μl of 1M7 (1-methyl-7-nitroisatoic anhydride) diluted in DMSO (final concentrations tested: 1, 2 and 4 mM). Modified RNAs were extracted by phenol/ chloroform and precipitated with Na-acetate and ethanol, in presence of 10 μg yeast RNA as carrier. After centrifugation, precipitates were dissolved in 4 μl of water, and reverse transcribed using radiolabeled primer ced267 (Supplementary Table S2) and Superscript IV reverse transcriptase (see Toeprint assays).

## RESULTS

### A point mutation in the +42 *tisB* mRNA 5′ stem-loop impairs 30S initiation complex formation and ribosome or S1 binding

**Figure 2.**
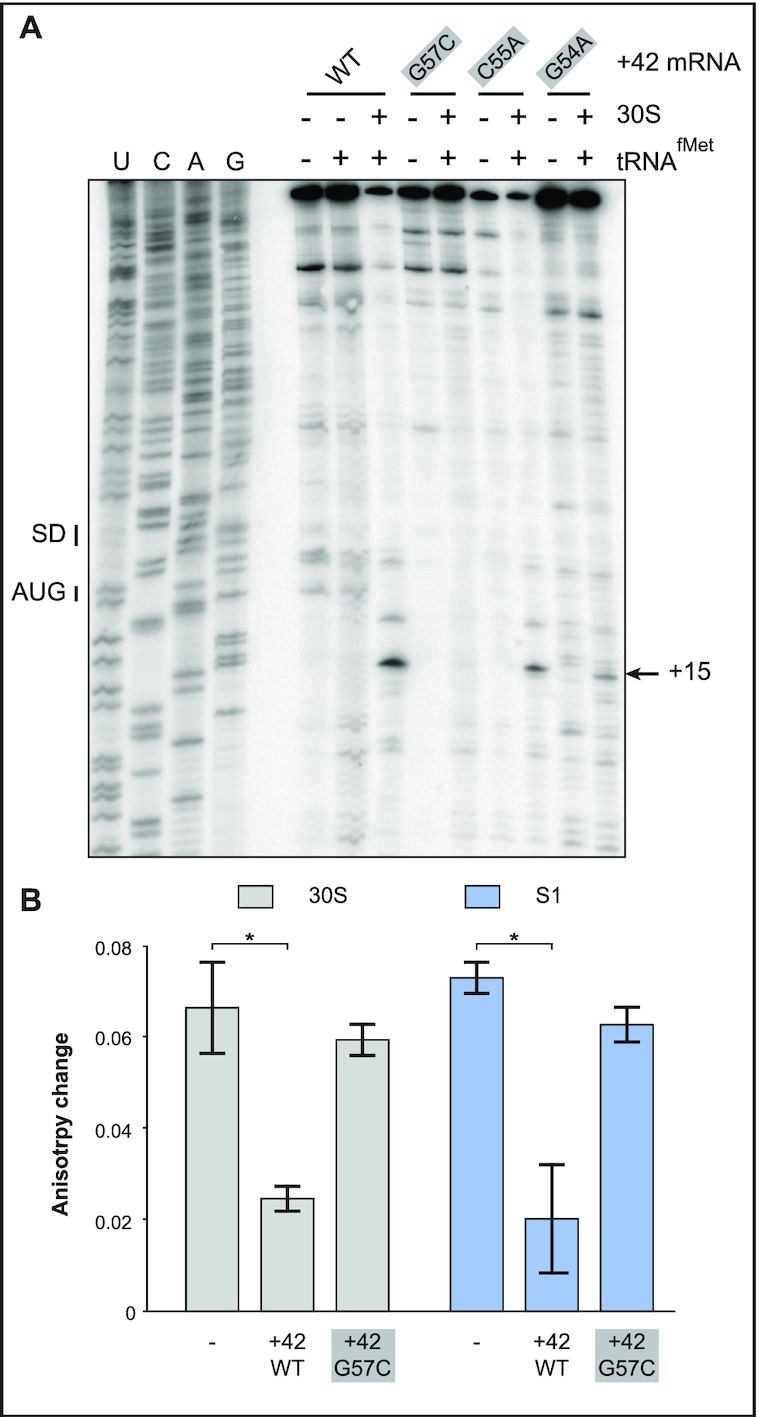
Effects of point mutations in the 5′end stem–loop on 30S-IC formation and ribosome/ S1 binding. (**A**) 30S-IC complex formation was monitored on +42 *tisB* mRNA (WT) and three mutant mRNAs with 5′ apical loop mutations (see Figure 1C). Toeprint signals at position 15, the signature of 30S-IC complex formation at the *tisB* RBS (SD and AUG – black bars), are indicated by a black arrow. (**B**) Fluorescence anisotropy change of 5′-end FAM-labeled +42 *tisB* mRNA in presence of a 10-fold molar excess of either 30S (grey columns) or purified protein S1 (blue). These complexes were challenged by a 50-fold molar excess of unlabeled wild-type +42 *tisB* mRNA or the corresponding G57C mutant variant. *: *P*-value < 0.05 (triplicate).

In a previous study, we addressed the molecular mechanisms of, and structural elements required for, standby-mediated translation of the translationally active +42 *tisB* mRNA (14). We showed that r-protein S1 promotes 30S ribosomal subunit binding to the single-stranded standby site of the mRNA. In addition, we identified a 5′ structural element that is essential for binding of the 30S subunit, and of purified S1 protein alone, to the standby site in the mRNA (14). Deletion of this element abolished standby. Furthermore, introduction of a stem breaker mutation (CC to AA substitutions at pos. 50 & 51) in the 5′ end stem-loop (pos. 50 to 64; Figure 1C) of +42 *tisB* mRNA drastically impaired ribosome binding and formation of the 30S-IC (14). Building on these results and the known binding preferences of S1 for RNA pseudoknots, we speculated that the 5′ terminal stem-loop could be part of an RNA pseudoknot. If this were the case, its loop sequence should base-pair to accessible flanking sequences, most likely nearby, and loop mutations would be expected to give phenotypes similar to a stem-breaker mutation (14). To assess this possibility, we used the HotKnots software (44–47) to predict candidate RNA pseudoknot structures in a 50 nt window upstream of the IstR1 base-pairing site in the 5′ end region of +42 *tisB* mRNA. Of 19 predicted structures (of Δ*G* < –6 kcal/mol), 13 were pseudoknots (Supplementary Table S3). Interestingly, the most stable pseudoknot predicted (Δ*G* = –9.53 kcal/mol; S1 = 9 nt, S2 = 5 nt, L1 = 0 nt, L2 = 2 nt, L3 = 3 nt), suggests single-stranded nucleotides 73–77 to be base-paired to loop nucleotides 53–57 to form the S2 stem (Figure 1C and D). In this model, substitutions G to A at position 54 (G54A), C to A at position 55 (C55A), and G to C at position 57 (G57C) should prevent formation of the pseudoknot and thus translation of +42 *tisB* mRNA (Figure 1E). These three independent mutations were introduced into +42 *tisB* mRNA, and 30S-IC formation tested by toeprint assays in comparison to wild-type +42 *tisB* mRNA as control. As expected, a strong toeprint signal at position +15 relative to the AUG start codon was observed with the wild type +42 *tisB* mRNA when incubated with both 30S subunits and initiator tRNA (Figure 2A) (11,14). In contrast, all three loop mutants showed impaired 30S-IC formation. While weaker toeprint signals were observed with the G54A and C55A mutants (8- and 1.5-fold decrease, respectively), the signal for the G57C mutant mRNA was virtually absent (Figure 2A). This suggested that the loop nucleotides in the 5′ stem-loop may be involved in a higher order RNA structure whose formation is essential for standby-mediated translation of +42 *tisB* mRNA.

We previously reported that mutations or truncations in the 5′end of the +42 *tisB* mRNA that abolished translation were correlated with decreased affinity of mutant mRNAs for the 30S subunit as well as S1 alone (14). We therefore tested +42 mRNA carrying the mutation with the strongest effect on toeprinting, G57C, for binding by using fluorescence anisotropy (FA) measurements. This method uses polarized light to excite a fluorophore, FAM, attached to the 5′ end of the +42 *tisB* mRNA, and records emitted light in planes both parallel and perpendicular to the plane of excitation. The read-out — change in anisotropy—informs on rotational diffusion, which in turn depends on whether a ligand (here: S1 or the 30S subunit) is bound. This method is explained in detail in Supplementary Figure S3A, and has previously allowed us to monitor 30S binding to the *tisB* mRNA standby site in absence of initiator tRNA (11,14). FA measures interaction of RNA-protein complexes in solution and does not require fractionation of complexes and free RNAs as in EMSA or filter binding assays.

Changes in fluorescence anisotropy were measured using a 5′-end FAM-labeled mRNA incubated with 30S subunits (14). This complex was then challenged with either a 50-fold molar excess of unlabeled competitor wild-type +42 *tisB* mRNA or the G57C mutant mRNA (see Supplementary Figure S3B). Control experiments in absence of competitor gave a high change in fluorescence anisotropy, i.e. the signature of 30S binding to the standby-competent and translationally active 5′end-FAM labeled +42 mRNA (Figure 2B). Addition of unlabeled +42 *tisB* mRNA strongly reduced the fluorescence anisotropy change, demonstrating that the 30S subunit relocated from the labeled to the unlabeled +42 mRNA in excess (Figure 2B). In contrast, high changes in fluorescence anisotropy were still obtained when the 30S/5′FAM +42 mRNA complex was challenged with the G57C mutant mRNA (Figure 2B). This shows that the single loop base substitution impaired the mRNA’s affinity for the 30S ribosome, in line with its effect on toeprinting (Figure 2A). Since S1 is required for standby translation on *tisB* mRNA, the same experimental design was used with purified S1 protein alone. As for 30S, unlabeled wild-type +42 mRNA effectively competed S1 binding to the 5′-end FAM-labeled mRNA, whereas the G57C mutant mRNA was ineffective (Figure 2B).

Taken together, the toeprint and binding assay results indicate that the loop nucleotides in the 5′-end hairpin of the +42 mRNA are required for standby, consistent with a requirement for a complex RNA element, such as a pseudoknot.

### The RNA pseudoknot structure constrains flexibility of the 5′UTR in the +42 *tisB* mRNA

Disruption of the putative pseudoknot by the most severe G57C substitution should be detectable by structure mapping. We therefore used SHAPE (selective 2′-hydroxyl acylation analyzed by primer extension) (48) to probe the differences between the 5′-UTR regions of wild-type and G57C mRNAs *in vitro* (Materials and Methods). Additional details of the method, and the experimental data obtained, are shown in Supplementary Figure S4A-C. The SHAPE results indeed support lower flexibility in the sequences encompassing the putative pseudoknot of wild-type +42 mRNA than of the G57C variant, in line with a disruption of local structure by the mutation. By contrast, SHAPE reactivities were unchanged in the standby region downstream (pos. 93 to 116), which is known to be single-stranded based on earlier chemical and enzymatic probing (11,14) (Supplementary Figure S4). Thus, structure probing combined with *in silico* prediction tentatively strengthens the pseudoknot prediction in Figure 1D.

### Overexpression of an mRNA with a G57C substitution is non-toxic *in vivo*

**Figure 3.**
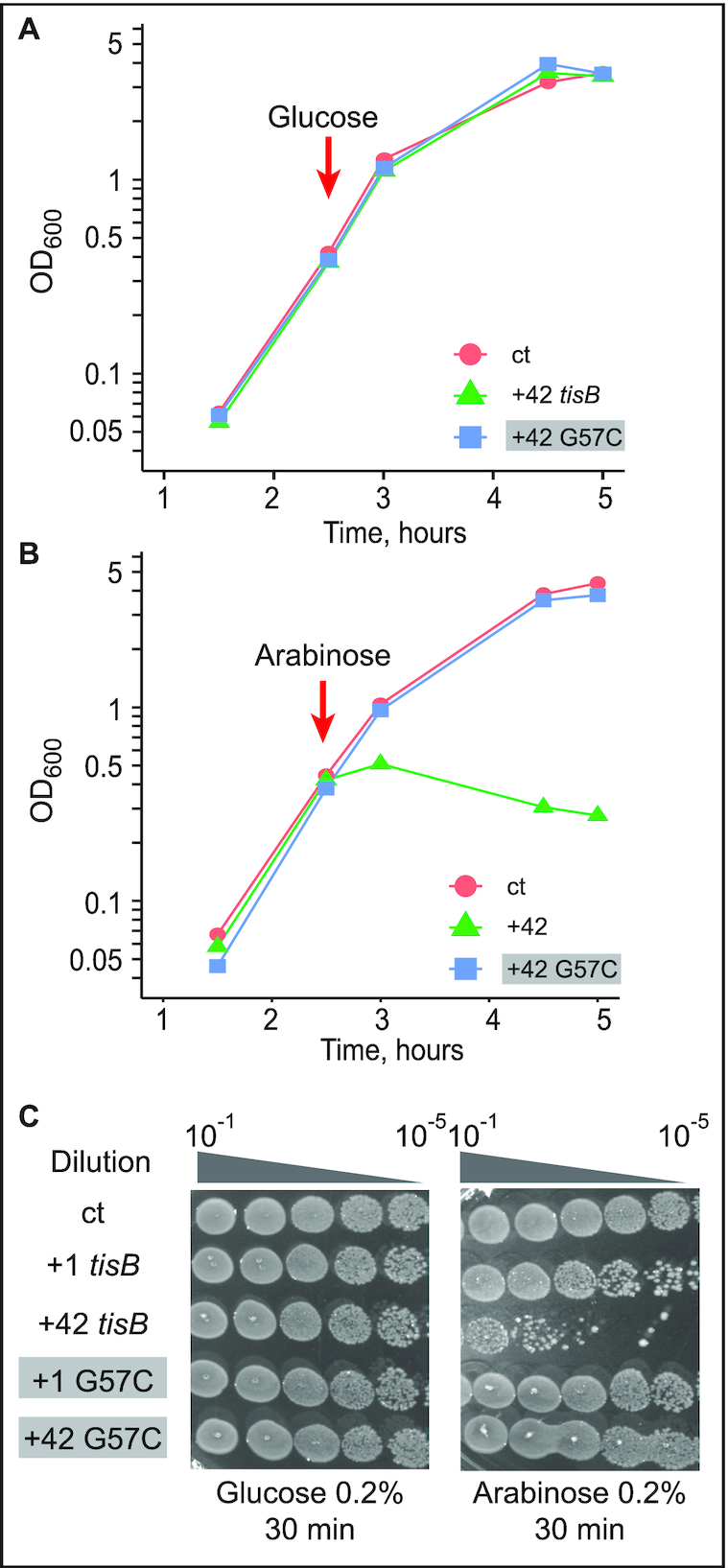
Toxicity in cells upon induction of wild-type or G57C mutant *tisB* mRNA. (**A**) *E. coli* cells carrying the plasmids pBAD control (ct; empty vector control), pBAD +42, or pBAD +42 G57C, were grown to mid-exponential phase (OD_600_ = 0.5) before addition of 0.2% glucose. Time of glucose addition is indicated by a red arrow. (**B**) Same protocol as in (A), but with induction of mRNA transcription by 0.2% arabinose. (**C**) Ten-fold serial dilutions of bacterial cultures 30 min post-induction by either glucose (left) or arabinose (right) were spotted on LA-amp plates. As indicated, spotting assays were also done with plasmids encoding delayed-active +1 *tisB* mRNA (see Results).

TisB depolarizes the inner membrane and, when overexpressed, triggers growth arrest and cell death (4,7). We used plasmids expressing +42 *tisB* mRNA under the control of an arabinose-inducible promoter, and created a variant carrying the G57C base substitution. Cells harboring the empty vector control (ct), and plasmids expressing either wild-type or G57C mutant mRNA, were grown to mid-exponential growth phase, followed by addition of either glucose (control, uninduced plasmids) or arabinose (toxin mRNA induction). No noticeable growth defect was observed in the presence of glucose (Figure 3A), as expected in the absence of induced mRNA transcription (confirmed by Northern blot analysis, Supplementary Figure S5A). In contrast, arabinose induction of wild-type mRNAs resulted in immediate growth arrest in the case of +42 *tisB* mRNA, whereas the G57C mutant +42 mRNA was non-toxic and without any observable growth effect, although it was expressed at similar levels as wild-type +42 mRNA (Figure 3B, Supplementary Figure S5A).

We next evaluated the effect of wild-type and mutant mRNAs on cell viability by spotting serial dilutions of bacterial cultures 30 min post-induction on LA-Amp plates. Here, we additionally included constructs that generated +1 mRNAs; wild-type +1 mRNA is delayed-toxic since processing is required to generated the active +42 mRNA (4,7). As in the growth curve analysis (Figure 3A), glucose caused no growth effects (Figure 3C). Spotting assays carried out after arabinose induction indicated that +1 and +42 *tisB* mRNA expression drastically affected cell viability by 100 and 10 000-fold, respectively (Figure 3C), whereas viability remained unaffected when plasmids encoded +1 G57C or +42 G57C mRNAs. Thus, growth curves and cell viability assays recapitulate the known toxic effects of the wild-type mRNAs (+1 and +42). However, induction of the G57C mutant mRNA gave no toxicity/lethality even when overexpressed from a high-copy number plasmid. We can conclude that the G57C mutation abolishes S1/30S binding, translation initiation complex formation and toxicity *in vivo*.

### A compensatory mutation in conjunction with G57C restores 30S initiation complex formation, 30S/ S1 binding, and *in vivo* toxicity

**Figure 4.**
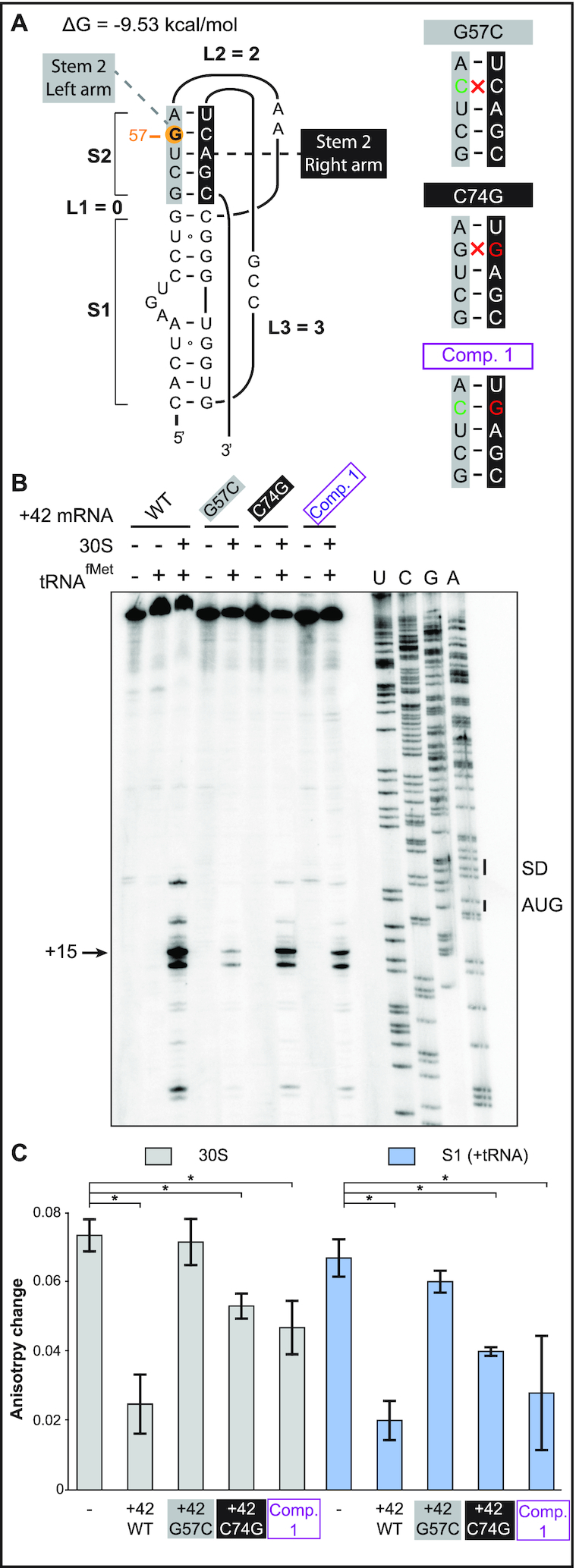
Rescue of 30S-IC formation and ribosome binding by the double-mutant G57C / C74G. (**A**) (Left) Secondary structure model of the 5′-end pseudoknot in wild-type +42 *tisB* mRNA. Nucleotides forming stem 2 are indicated in grey and black boxes (left and right arms of the helix, respectively), and the G57 is highlighted by an orange circle. (Right) Representation of the stem 2 helix with the G57C base substitution (green base), C74G (red) and the double compensatory mutant Comp. 1 (purple). Disruption of Watson-Crick base pairing is indicated (red cross). (**B**) Toeprint assays on +42 *tisB* mRNA (WT) and mutated variants G57C, C74G, and the compensatory double mutant G57C/C74G (Comp. 1). The position of the toeprint signal at +15 is indicated. (**C**) Fluorescence anisotropy change of 5′-end FAM-labeled +42 *tisB* mRNA was conducted as in Figure 2B. Complexes were competed with a 50-fold molar excess of wild-type +42 *tisB* mRNA or the corresponding G57C, C74G, and G57C/C74G (Comp. 1) variants. *P-value < 0.05 (triplicate)

Point mutations in the 5′ end stem-loop, either in the stem or in the apical loop region, severely impaired *tisB* mRNA translation both *in vitro* and *in vivo* (Figures 2 and 3; (14)). Based on the pseudoknot prediction model in Figure 1D, a C to G substitution at position 74 should restore the five base-pair helix if combined with the G57C mutation in stem 2 of the pseudoknot (Figures 1C, D and 4A; for clarity, the left arm of the stem is boxed in grey, and the right arm in black, throughout the paper). Therefore, we designed two additional *tisB* mRNA mutants, C74G and the double (compensatory) G57C/C74G (Comp. 1, Figure 4A), and tested performance by toeprinting. For comparison, we used wild-type +42 *tisB* mRNA (positive control) and the G57C mutant mRNA (negative control). Again, the G57C mutant mRNA gave a barely detectable toeprint signal (≈20 fold lower compared to wild-type mRNA) (Figure 4B). The point mutation C74G alone, predicted to weaken or abolish pseudoknot formation, gave a detectable toeprint signal (1.8-fold reduction compared to wild-type mRNA) (see Discussion), and the double mutation G57C/C74G significantly improved 30S-IC formation over the single G57C, yet without reaching wild-type levels (4-fold lower than +42 mRNA; Figure 4B). These results suggest that the disruption of the proposed pseudoknot in the G57C mutant mRNA can be rescued by introducing a compensatory mutation matching the pseudoknot structure prediction.

To validate this result, competition assays with 5-′end FAM-labeled +42 mRNA were done as in Figure 2. Again, wild-type unlabeled +42 mRNA efficiently competed both 30S and protein S1 binding, whereas the G57C mutant mRNA failed to do so (Figure 4C and Supplementary Figure S6). Congruent with the toeprint results, combining the compensatory C74G mutation with G57C restored an efficient competitor capable of challenging both 30S subunit and S1 binding to the labeled mRNA (Figure 4C). Surprisingly, the C74G mutant alone was also an efficient competitor, in line with the toeprint assay (Figure 4B). These *in vitro* results indicate that the defects in ribosome binding and translation of the G57C mutant mRNA can be rescued by the compensatory base substitution C74G.

**Figure 5.**
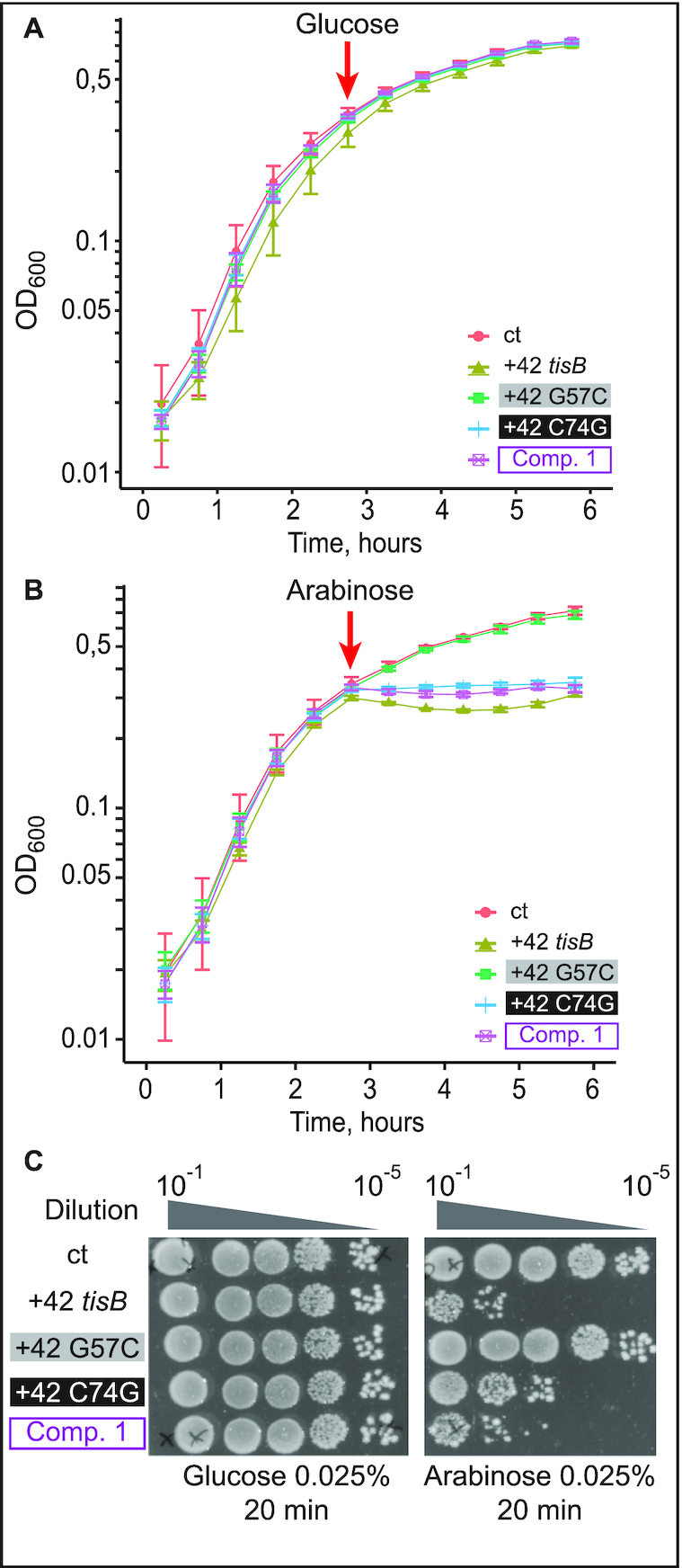
Expression of the compensatory double-mutant G57C/C74G, but not of G57C, is lethal *in vivo*. (**A**) *E. coli* cells carrying pBAD plasmids as indicated were grown to OD_600_ = 0.5 in a Tecan plate reader at 37°C before addition of 0.025% glucose. Time of addition is indicated (red arrow). (**B**) Same as in (A), but with arabinose induction (0.025%). (**C**) Ten-fold serial dilutions of bacterial cultures 30 min post-induction with either glucose or arabinose were spotted on LA-amp plates.

To address whether the G57C/C74G double mutant confers toxicity to the *tisB* mRNA *in vivo*, assays were carried out as before (Figure 3), with the C74G substitution introduced into either the wild-type +42 or the +42 G57C mutant mRNA. Expression of the mRNAs was induced at OD_600_ = 0.5 with either glucose (negative control) or arabinose, followed by growth measurements and spotting assays. As in Figure 3, the empty vector control failed to cause toxicity in presence of arabinose as did the G57C mutant (Figure 5A), even though *tisB* mRNA was expressed from the plasmid (Supplementary Figure S5B). Again, expression of wild-type +42 *tisB* mRNA stopped growth (Figure 5B). Interestingly, induction of the G57C/C74G double mutant (Comp. 1) mRNA gave similar lethality as wild-type +42 mRNA, both in liquid and on solid media (Figure 5B, C). Expression of the C74G single mutant mRNA arrested growth to a degree comparable to its wild-type counterpart in liquid medium (Figure 5B). However, spotting assays indicated 10-fold less toxicity compared to the wild-type +42 or the double mutant G57C/C74G (Figure 5C). Northern blot analysis confirmed that all mRNAs were expressed solely upon arabinose induction (Supplementary Figure S5B).

### The effects of double nucleotide mutations strongly support the RNA pseudoknot predicted in the 5′UTR of +42 *tisB* mRNA

**Figure 6.**
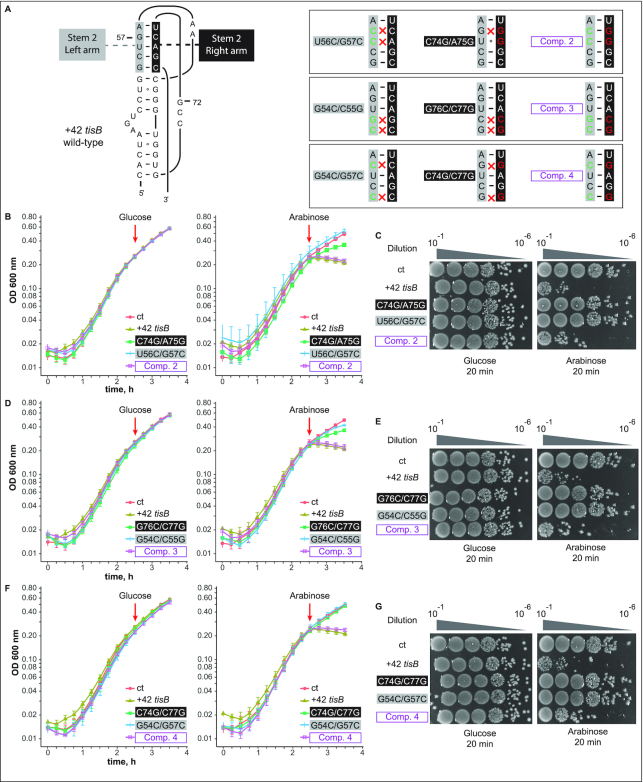
Double base substitutions in either side of the pseudoknot stem 2 impair toxicity, while compensatory mutants are lethal *in vivo*. (**A**) (Left) Schematics of the pseudoknot, for comparison. (Right) Boxes show stem 2 only, using the same color code as on the left. Double nucleotide substitutions on each arm, and compensatory quadruple mutations (purple) are indicated. (**B**) *E. coli* cells carrying an empty vector control (ct) or pBAD plasmids expressing the wild-type +42 *tisB* mRNA, or mRNAs with the U56C/G57C, C74G/A75G or Comp. 2 mutation, were grown to OD_600_ = 0.3 in a Tecan plate reader before addition (red arrow) of 0.025% glucose (left) or arabinose (right). All experiments in B, D, F were conducted four times. (**C**) Ten-fold serial dilutions of bacterial cultures (same strains as in B) were subjected to plating assays as in Figure 5C. (**D, E**). Same as (B, C) but with cells carrying pBAD plasmids expressing U56C/G57C, C74G/A75G, or Comp. 3 +42 *tisB* mRNAs. (**F**, **G**). Same as (B, C) but with cells carrying pBAD plasmids expressing G54/G57, C74G/C77G, or Comp. 4 +42 *tisB* mRNAs.

Even though the compensatory mutation G57C/C74G rescued the lack of toxicity observed with G57C alone, the C74G single mutant unexpectedly remained toxic (Figure 5B and C), and thus raised possible alternative explanations. To rigorously test the pseudoknot model, we therefore created three additional sets of mutations, each of which changes two nucleotides each in either the left or right arm of stem 2, or were combined to restore base-pairing. These mutations are shown on the pseudoknot structure in Figure 6A. First, a second base substitution, U56C, was combined with the G57C mutation to further destabilize stem 2 (U56C/G57C), and additional left arm mutants were G54C/C55G and G54C/G57C (Figure 6A). G54C/C55G was chosen because mutations in these two positions individually already impacted toeprinting signals (see Figure 2). The second set of two nt-change mutants were the corresponding (right arm) complements C74G/A75G, G76C/C77G and C74G/C77G. Finally, Comp 2, 3, and 4 were the quadruple mutations designed to restore stem 2 base-pairing in the three cases (Figure 6A).

Toxicity assays were performed as in Figure 5 with plasmid constructs expressing the panel of all these mutant +42 *tisB* mRNAs. TisB-mediated toxicity was monitored again in liquid culture and on solid media (Figure 6B-G). No noticeable growth defect was observed in the presence of glucose (Figure 6B, D, F). Disruption of the left side of stem 2: U56C/G57C (Figure 6B and C), G54C/C55G (Figure 6D and E), G54C/G57C (Figure 6F and G) abolished TisB-mediated toxicity both in liquid and on solid medium. Similarly, and unlike the effect of the C74G single mutant (Figure 5B and C), all double base substitutions on the right side of stem 2, C74G/A75G (Figure 6B and C), G76C/C77G (Figure 6D and E) and C74G/C77G (Figure 6F and G), were non-toxic. Strikingly, all compensatory mutations combining changes on both sides of stem 2 gave toxicity levels similar to that of the wild-type +42 *tisB* mRNA (Comp. 2–4; Figure 6B–G). Taken together, these results lend strong support for the presence of the RNA pseudoknot structure at the 5′end of the +42 *tisB* mRNA and its essentiality for translation of the toxin.

## DISCUSSION

In this paper, we present *in vitro* and *in vivo* evidence that the apical loop residues of the 5′ terminal hairpin of the +42 *tisB* mRNA are involved in the formation of a pseudoknot structure which is essential for the functionality of the mRNA. First, we showed that three individual base substitutions in the apical loop impaired formation of the 30S-IC compared to wild-type +42 mRNA (Figure 2A). One of these, G57C, virtually abolished toeprint formation (Figures 2A and 4B). Fluorescence anisotropy experiments showed that this mutant mRNA had a decreased affinity for both 30S and S1 binding (Figure 2B), tentatively explaining the observed effects on 30S-IC formation. Plasmids overexpressing the *tisB* mRNA carrying the G57C mutation conferred a complete absence of toxic effects (Figure 3). We then created a point mutation, C74G, that compensated 30S-IC formation, 30S/S1 binding, and toxicity to the +42 G57C *tisB* mRNA (Figures 4 and 5). These mutations, G57C and C74G, matched the prediction of a stable pseudoknot (Δ*G* = –9.53 kcal/mol), obtained with HotKnots and PseudoViewer software (Figures 1D, 4A, Supplementary Table S3). Moreover, six double base substitutions disrupting either side of stem 2 (U56C/G57C, G54C/C55G, G54/G57, C74G/A75G, G76C/C77G, C74G/C77G) resulted in a complete loss of toxicity, whereas all three compensatory mutants (Comp. 2, 3, and 4)—designed to restore the pseudoknot structure (Figure 6A)—conferred toxicity identical (Comp 2 and 3; Figure 6B–E), or similar (Figure 6F and G), to wild-type +42 mRNA. The pseudoknot model that best matches both predicted and empirical data is of type H (S1 = 9 nt, S2 = 5 nt, L1 = 0 nt, L2 = 2 nt and L3 = 3 nt) (Figure 1D).

**Figure 7.**
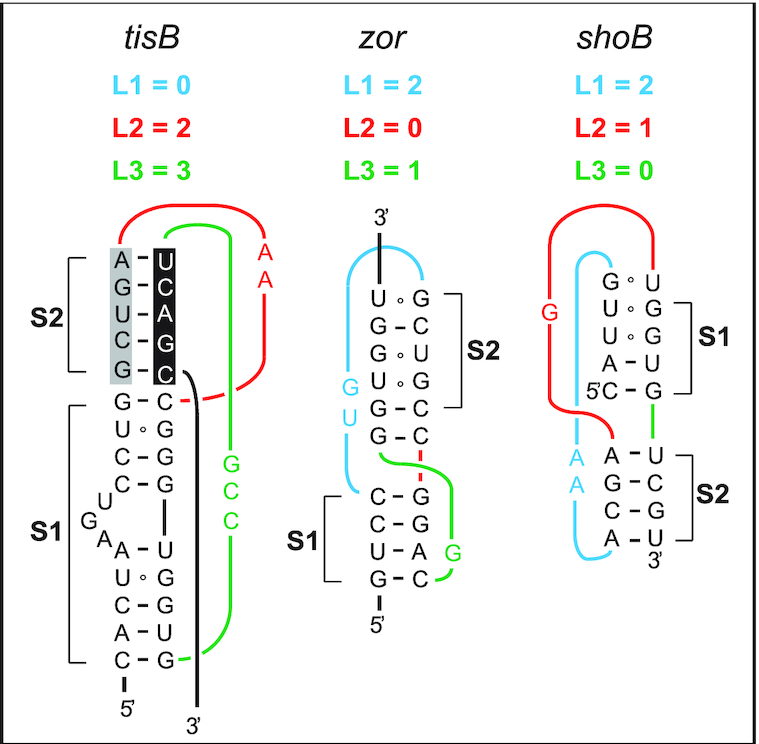
Predicted RNA pseudoknots in type I TA systems in *E. coli*. Predicted RNA pseudoknots at equivalent positions near the 5′-ends of three toxin mRNAs are shown (see Discussion).

Could pseudoknots be a more general feature of toxin mRNAs? Several type I TA toxin loci in *E. coli* are non-homologous but share strikingly similar regulatory features (49). Like in the case of *tisB*, the *zor* toxin gene from the TA system *zor-orz*, and *shoB* from *shoB-ohsC*, are first transcribed into stably structured and translationally inactive mRNAs (50,51). Like for +42 *tisB* mRNA, nucleolytic cleavages generate 5′-truncated, translationally active mRNAs, denoted ‘+28’ for *zor* and ‘+40’ for *shoB* (51,52). Finally, like IstR1, the antitoxin RNAs Orz and OhsC inhibit translation of their cognate toxin mRNAs by base-pairing far upstream of the toxin RBS, suggesting a standby-dependent model of translational regulation (49,52,53). Based on these similarities, and strengthened by the observation that 5′-structures are required for standby activity for *tisB* (14), we hypothesize that pseudoknot RNA structures may play the role suggested by the present results also in these other toxin mRNAs. Indeed, HotKnots software run on *tisB* mRNA, and the two toxin mRNAs *zor* and *shoB*, within a 50 nt window preceding the annotated base-pairing site of the antitoxin sRNAs, suggested converging features (10,45,52,53). As for *tisB*, pseudoknots dominate the predicted structures for both *zor* (nine pseudoknots out of 16 predictions) and *shoB* (two out of two structure predictions) (10,52,53). The data obtained suggest structural diversity in the predicted pseudoknot topologies, both in stems and connected loop lengths (see Figure 7 for the most stable examples, and Supplementary Table S3 for a complete list). We propose that all these active mRNAs may carry pseudoknot motifs at equivalent positions. Differences in pseudoknot topology are not surprising when considering the diversity of known S1-binding pseudoknots in general (Supplementary Figure S1) (26,36,37,40). Hence, it is tempting to speculate that the enterobacterial type I TA systems that rely on standby-dependent toxin translation share key features. Standby in *tisB* strictly requires S1 for proper anchoring of the 30S ribosome to the standby site and unwinding activity towards the toxin RBS, and this in turn is dependent on a 5′ structure element (14). We argue here that this structure conforms to a pseudoknot topology that creates high affinity for S1 binding, which might well play an equivalent role in the two other toxin mRNAs as detailed above.

It is worth mentioning that the definition of standby has become more complicated since the original model was proposed (13,54). For instance, the relationship between a translational enhancer and a standby site is not entirely clear, and may well be overlapping. Enhancers are often found in close vicinity of bacterial mRNAs’ RBSs and often stimulate translation by recruiting S1 (21,24,55). Standby sites can also act as translation enhancers. For instance, one study convincingly demonstrated that a standby region immediately upstream of an accessible, *not* structurally sequestered RBS in the *lpp* mRNA can kinetically boost the translation rate. In this case, this occurs because a standby-bound 30S is already present immediately upstream of the 70S ribosome residing at the RBS. Thereby, standby binding enables rapid re-initiation once the leading 70S enters elongation and departs from the RBS (56). Clearly, different translation-enhancing effects caused by standby or ‘enhancers’ may share common denominators, such as a prominent role of ribosomal protein S1 (21,24,55,57,58). However, cases like that of *tisB* mRNA are distinct in that they require standby to overcome RNA structure at a great distance, and where translation—in line with the original standby model—without standby is at background level (10–13,54). We argue here that, particularly in the cases where standby occurs far upstream of stably structured RBSs, a significantly long 30S residence time on the standby site is required. Based on our results, and suggested by comparisons to other TA toxin mRNAs that share the same regulatory pattern, we propose that 5′-pseudoknots together with nearby unstructured regions make up the two elements that together create the high affinity binding sites for S1 and/or 30S required for a significantly long dwelling time. This in turn would permit S1 to act as an ‘unwindase’ to promote progression of the 30S through the impeding structure—which is unique to systems in which standby occurs at a great distance—to ultimately access the toxin RBS. Clearly, it will be of great interest to obtain high-resolution structures of 5′-segments of *tisB* mRNA in order to substantiate the proposed model.

## Supplementary Material

gkaa1139_Supplemental_File

## Notes

Present address: Anne Lippegaus, Institute of Microbiology, Friedrich-Schiller-University Jena, 07745 Jena, Germany.

## References

[1] Lewis L.K., Harlow G.R., Gregg-Jolly L.A., Mount D.W. Identification of high affinity binding sites for LexA which define new DNA damage-inducible genes in *Escherichia coli*. J. Mol. Biol. 1994; 241:507–523. 8057377 10.1006/jmbi.1994.1528

[2] Courcelle J., Khodursky A., Peter B., Brown P.O., Hanawalt P.C. Comparative gene expression profiles following UV exposure in wild-type and SOS-deficient *Escherichia coli*. Genetics. 2001; 158:41–64. 11333217 10.1093/genetics/158.1.41 PMC1461638

[3] Fernández De Henestrosa A.R., Ogi T., Aoyagi S., Chafin D., Hayes J.J., Ohmori H., Woodgate R. Identification of additional genes belonging to the LexA regulon in *Escherichia coli*. Mol. Microbiol. 2000; 35:1560–1572. 10760155 10.1046/j.1365-2958.2000.01826.x

[4] Unoson C., Wagner E.G.H. A small SOS-induced toxin is targeted against the inner membrane in *Escherichia coli*. Mol. Microbiol. 2008; 70:258–270. 18761622 10.1111/j.1365-2958.2008.06416.x

[5] Dörr T., Vulić M., Lewis K. Ciprofloxacin causes persister formation by inducing the TisB toxin in *Escherichia coli*. PLoS Biol. 2010; 8:e1000317. 20186264 10.1371/journal.pbio.1000317 PMC2826370

[6] Berghoff B.A., Karlsson T., Källman T., Wagner E.G.H., Grabherr M.G. RNA-sequence data normalization through *in silico* prediction of reference genes: the bacterial response to DNA damage as case study. BioData Min. 2017; 10:30. 28878825 10.1186/s13040-017-0150-8 PMC5584328

[7] Berghoff B.A., Hoekzema M., Aulbach L., Wagner E.G.H. Two regulatory RNA elements affect TisB-dependent depolarization and persister formation. Mol. Microbiol. 2017; 103:1020–1033. 27997707 10.1111/mmi.13607

[8] Stapels D.A.C., Hill P.W.S., Westermann A.J., Fisher R.A., Thurston T.L., Saliba A.-E., Blommestein I., Vogel J., Helaine S. Salmonella persisters undermine host immune defenses during antibiotic treatment. Science. 2018; 362:1156–1160. 30523110 10.1126/science.aat7148

[9] Gollan B., Grabe G., Michaux C., Helaine S. Bacterial persisters and infection: Past, present, and progressing. Annu. Rev. Microbiol. 2019; 73:359–385. 31500532 10.1146/annurev-micro-020518-115650

[10] Vogel J., Argaman L., Wagner E.G.H., Altuvia S. The small RNA IstR inhibits synthesis of an SOS-induced toxic peptide. Curr. Biol. 2004; 14:2271–2276. 15620655 10.1016/j.cub.2004.12.003

[11] Darfeuille F., Unoson C., Vogel J., Wagner E.G.H. An antisense RNA inhibits translation by competing with standby ribosomes. Mol. Cell. 2007; 26:381–392. 17499044 10.1016/j.molcel.2007.04.003

[12] Sterk M., Romilly C., Wagner E.G.H. Unstructured 5′-tails act through ribosome standby to override inhibitory structure at ribosome binding sites. Nucleic Acids Res. 2018; 46:4188–4199. 29420821 10.1093/nar/gky073 PMC5934652

[13] de Smit M.H., van Duin J. Translational standby sites: how ribosomes may deal with the rapid folding kinetics of mRNA. J. Mol. Biol. 2003; 331:737–743. 12909006 10.1016/s0022-2836(03)00809-x

[14] Romilly C., Deindl S., Wagner E.G.H. The ribosomal protein S1-dependent standby site in *tisB* mRNA consists of a single-stranded region and a 5′ structure element. Proc. Natl. Acad. Sci. U.S.A. 2019; 116:15901–15906. 31320593 10.1073/pnas.1904309116 PMC6690012

[15] Qu X., Lancaster L., Noller H.F., Bustamante C., Tinoco I. Ribosomal protein S1 unwinds double-stranded RNA in multiple steps. Proc. Natl. Acad. Sci. U.S.A. 2012; 109:14458–14463. 22908248 10.1073/pnas.1208950109 PMC3437903

[16] Rajkowitsch L., Schroeder R. Dissecting RNA chaperone activity. RNA. 2007; 13:2053–2060. 17901153 10.1261/rna.671807 PMC2080586

[17] Sørensen M.A., Fricke J., Pedersen S. Ribosomal protein S1 is required for translation of most, if not all, natural mRNAs in *Escherichia coli in vivo*. J. Mol. Biol. 1998; 280:561–569. 9677288 10.1006/jmbi.1998.1909

[18] Subramanian A.-R., van Duin J. Exchange of individual ribosomal proteins between ribosomes as studied by heavy isotope-transfer experiments. Molec. Gen. Genet. 1977; 158:1–9. 342903 10.1007/BF00455113

[19] Suryanarayana T., Subramanian A.R. An essential function of ribosomal protein S1 in messenger ribonucleic acid translation. Biochemistry. 1983; 22:2715–2719. 6307346 10.1021/bi00280a020

[20] Hajnsdorf E., Boni I.V. Multiple activities of RNA-binding proteins S1 and Hfq. Biochimie. 2012; 94:1544–1553. 22370051 10.1016/j.biochi.2012.02.010

[21] Komarova A.V., Tchufistova L.S., Dreyfus M., Boni I.V. AU-Rich sequences within 5′ untranslated leaders enhance translation and stabilize mRNA in *Escherichia coli*. J. Bacteriol. 2005; 187:1344–1349. 15687198 10.1128/JB.187.4.1344-1349.2005 PMC545611

[22] Cifuentes-Goches J.C., Hernández-Ancheyta L., Guarneros G., Oviedo N., Hernández-Sánchez J. Domains two and three of *Escherichia coli* ribosomal S1 protein confers 30S subunits a high affinity for downstream A/U-rich mRNAs. J. Biochem. 2019; 166:29–40. 30668774 10.1093/jb/mvz006

[23] Hook-Barnard I.G., Brickman T.J., McIntosh M.A. Identification of an AU-rich translational enhancer within the *Escherichia coli fepB* leader RNA. J. Bacteriol. 2007; 189:4028–4037. 17400738 10.1128/JB.01924-06 PMC1913407

[24] Azam M.S., Vanderpool C.K. Translation inhibition from a distance: The small RNA SgrS silences a ribosomal protein S1-dependent enhancer. Mol. Microbiol. 2020; 114:391–408. 32291821 10.1111/mmi.14514 PMC7502529

[25] Boni I.V., Artamonova V.S., Tzareva N.V., Dreyfus M. Non‐canonical mechanism for translational control in bacteria: synthesis of ribosomal protein S1. EMBO J. 2001; 20:4222–4232. 11483525 10.1093/emboj/20.15.4222 PMC149162

[26] Duval M., Korepanov A., Fuchsbauer O., Fechter P., Haller A., Fabbretti A., Choulier L., Micura R., Klaholz B.P., Romby P. et al. *Escherichia coli* ribosomal protein S1 unfolds structured mRNAs onto the ribosome for active translation Initiation. PLoS Biol. 2013; 11:e1001731. 24339747 10.1371/journal.pbio.1001731 PMC3858243

[27] Kolb A., Hermoso J.M., Thomas J.O., Szer W. Nucleic acid helix-unwinding properties of ribosomal protein S1 and the role of S1 in mRNA binding to ribosomes. Proc. Natl. Acad. Sci. U.S.A. 1977; 74:2379–2383. 329281 10.1073/pnas.74.6.2379 PMC432175

[28] Qureshi N.S., Bains J.K., Sreeramulu S., Schwalbe H., Fürtig B. Conformational switch in the ribosomal protein S1 guides unfolding of structured RNAs for translation initiation. Nucleic Acids Res. 2018; 46:10917–10929. 30124944 10.1093/nar/gky746 PMC6237739

[29] Sacerdot C., Caillet J., Graffe M., Eyermann F., Ehresmann B., Ehresmann C., Springer M., Romby P. The *Escherichia coli* threonyl-tRNA synthetase gene contains a split ribosomal binding site interrupted by a hairpin structure that is essential for autoregulation. Mol. Microbiol. 1998; 29:1077–1090. 9767575 10.1046/j.1365-2958.1998.00995.x

[30] Hilbers C.W., Michiels P.J.A., Heus H.A. New developments in structure determination of pseudoknots. Biopolymers. 1998; 48:137–153. 10333742 10.1002/(SICI)1097-0282(1998)48:2<137::AID-BIP4>3.0.CO;2-H

[31] Peselis A., Serganov A. Structure and function of pseudoknots involved in gene expression control. Wiley Interdiscip. Rev. RNA. 2014; 5:803–822. 25044223 10.1002/wrna.1247 PMC4664075

[32] Westhof E., Jaeger L. RNA pseudoknots. Curr. Opin. Struct. Biol. 1992; 2:327–333.

[33] van Batenburg F.H.D., Gultyaev A.P., Pleij C.W.A. PseudoBase: structural information on RNA pseudoknots. Nucleic Acids Res. 2001; 29:194–195. 11125088 10.1093/nar/29.1.194 PMC29770

[34] Gultyaev A.P., Olsthoorn R.C., Pleij C.W., Westhof E. RNA structure: pseudoknots. eLS. 2012; doi:10.1002/9780470015902.a0003134.pub2.

[35] Han K., Byun Y. PseudoViewer2: visualization of RNA pseudoknots of any type. Nucleic Acids Res. 2003; 31:3432–3440. 12824341 10.1093/nar/gkg539 PMC168946

[36] Ringquist S., Jones T., Snyder E.E., Gibson T., Boni I., Gold L. High-affinity RNA ligands to *Escherichia coli* ribosomes and ribosomal protein S1: Comparison of natural and unnatural binding sites. Biochemistry. 1995; 34:3640–3648. 7534475 10.1021/bi00011a019

[37] Bordeau V., Felden B. Ribosomal protein S1 induces a conformational change of tmRNA; more than one protein S1 per molecule of tmRNA. Biochimie. 2002; 84:723–729. 12457560 10.1016/s0300-9084(02)01442-6

[38] Wower I.K., Zwieb C.W., Guven S.A., Wower J. Binding and cross-linking of tmRNA to ribosomal protein S1, on and off the *Escherichia coli* ribosome. EMBO J. 2000; 19:6612–6621. 11101533 10.1093/emboj/19.23.6612 PMC305868

[39] Wower I.K., Zwieb C., Wower J. Contributions of pseudoknots and protein SmpB to the structure and function of tmRNA in *trans*-translation. J. Biol. Chem. 2004; 279:54202–54209. 15494393 10.1074/jbc.M410488200

[40] Lund P.E., Chatterjee S., Daher M., Walter N.G. Protein unties the pseudoknot: S1-mediated unfolding of RNA higher order structure. Nucleic Acids Res. 2020; 48:2107–2125. 31832686 10.1093/nar/gkz1166 PMC7038950

[41] Hoekzema M., Romilly C., Holmqvist E., Wagner E.G.H. Hfq‐dependent mRNA unfolding promotes sRNA‐based inhibition of translation. EMBO J. 2019; 38:e101199. 30833291 10.15252/embj.2018101199 PMC6443205

[42] Moore M.J., Sharp P.A. Site-specific modification of pre-mRNA: the 2′-hydroxyl groups at the splice sites. Science. 1992; 256:992–997. 1589782 10.1126/science.1589782

[43] Akiyama B.M., Stone M.D. Chapter 2 - Assembly of complex RNAs by splinted ligation. Methods in Enzymology, Biophysical, Chemical, and Functional Probes of RNA Structure, Interactions and Folding: Part B. 2009; 469:Academic Press 27–46. 10.1016/S0076-6879(09)69002-9 20946783

[44] Ren J., Rastegari B., Condon A., Hoos H.H. HotKnots: Heuristic prediction of RNA secondary structures including pseudoknots. RNA. 2005; 11:1494–1504. 16199760 10.1261/rna.7284905 PMC1370833

[45] Andronescu M.S., Pop C., Condon A.E. Improved free energy parameters for RNA pseudoknotted secondary structure prediction. RNA. 2010; 16:26–42. 19933322 10.1261/rna.1689910 PMC2802035

[46] Han K., Lee Y., Kim W. PseudoViewer: automatic visualization of RNA pseudoknots. Bioinformatics. 2002; 18:S321–S328. 12169562 10.1093/bioinformatics/18.suppl_1.s321

[47] Byun Y., Han K. PseudoViewer3: generating planar drawings of large-scale RNA structures with pseudoknots. Bioinformatics. 2009; 25:1435–1437. 19369500 10.1093/bioinformatics/btp252

[48] Smola M.J., Rice G.M., Busan S., Siegfried N.A., Weeks K.M. Selective 2′-hydroxyl acylation analyzed by primer extension and mutational profiling (SHAPE-MaP) for direct, versatile, and accurate RNA structure analysis. Nat. Protoc. 2015; 10:1643–1669. 26426499 10.1038/nprot.2015.103 PMC4900152

[49] Fozo E.M. New type I toxin-antitoxin families from ‘wild’ and laboratory strains of *E. coli*: Ibs-Sib, ShoB-OhsC and Zor-Orz. RNA Biol. 2012; 9:1504–1512. 23182878 10.4161/rna.22568

[50] Kawano M., Reynolds A.A., Miranda-Rios J., Storz G. Detection of 5′- and 3′-UTR-derived small RNAs and cis-encoded antisense RNAs in *Escherichia coli*. Nucleic Acids Res. 2005; 33:1040–1050. 15718303 10.1093/nar/gki256 PMC549416

[51] Wen J., Harp J.R., Fozo E.M. The 5′ UTR of the type I toxin ZorO can both inhibit and enhance translation. Nucleic Acids Res. 2017; 45:4006–4020. 27903909 10.1093/nar/gkw1172 PMC5397157

[52] Fozo E.M., Kawano M., Fontaine F., Kaya Y., Mendieta K.S., Jones K.L., Ocampo A., Rudd K.E., Storz G. Repression of small toxic protein synthesis by the Sib and OhsC small RNAs. Mol. Microbiol. 2008; 70:1076–1093. 18710431 10.1111/j.1365-2958.2008.06394.x PMC2597788

[53] Wen J., Won D., Fozo E.M. The ZorO-OrzO type I toxin–antitoxin locus: repression by the OrzO antitoxin. Nucleic Acids Res. 2014; 42:1930–1946. 24203704 10.1093/nar/gkt1018 PMC3919570

[54] de Smit M.H., van Duin J. Secondary structure of the ribosome binding site determines translational efficiency: a quantitative analysis. Proc. Natl. Acad. Sci. U.S.A. 1990; 87:7668–7672. 2217199 10.1073/pnas.87.19.7668 PMC54809

[55] Aseev L.V., Koledinskaya L.S., Boni I.V. Autogenous regulation *in vivo* of the *rpmE* gene encoding ribosomal protein L31 (bL31), a key component of the protein–protein intersubunit bridge B1b. RNA. 2020; 26:814–826. 32209634 10.1261/rna.074237.119 PMC7297116

[56] Andreeva I., Belardinelli R., Rodnina M.V. Translation initiation in bacterial polysomes through ribosome loading on a standby site on a highly translated mRNA. Proc. Natl. Acad. Sci. U.S.A. 2018; 115:4411–4416. 29632209 10.1073/pnas.1718029115 PMC5924895

[57] Komarova A.V., Tchufistova L.S., Supina E.V., Boni I.V. Protein S1 counteracts the inhibitory effect of the extended Shine-Dalgarno sequence on translation. RNA. 2002; 8:1137–1147. 12358433 10.1017/s1355838202029990 PMC1370328

[58] Takahashi S., Furusawa H., Ueda T., Okahata Y. Translation enhancer improves the ribosome liberation from translation initiation. J. Am. Chem. Soc. 2013; 135:13096–13106. 23927491 10.1021/ja405967h

