# Supplementary material for: An RNA pseudoknot is essential for standby-mediated translation of the tisB toxin mRNA in Escherichia coli
Source: Nucleic Acids Res. 2020 Nov 24;48(21):12336–47. doi: 10.1093/nar/gkaa1139 (PMC7708055; doi:10.1093/nar/gkaa1139)
Supplement: gkaa1139_Supplemental_File [file gkaa1139_supplemental_file.pdf]

Supplementary Material

An RNA pseudoknot is essential for standby-mediated translation of the *tisB* toxin mRNA in *Escherichia coli*

Cédric Romilly[1,*], Anne Lippegaus[1,#], and E. Gerhart H. Wagner[1,*]

[1] Department of Cell and Molecular Biology, Uppsala University, S-75124, Sweden

*To whom correspondence should be addressed. C. Romilly Tel: +46 184714470; Fax: +46 18530396; Email: cedric.romilly@gmail.com. E.G.H Wagner Tel: +46 184714866; Fax: +46 18530396; Email: gerhart.wagner@icm.uu.se

#Present address: Institute of Microbiology, Friedrich-Schiller-University Jena, 07745 Jena, Germany

Table S1: Plasmids and strains used in this study

Name	Strain/ Genotype	Plasmid (a)	Antibiotic marker	Used for (a)	Ref.
ced3	MG1655	pJV 974-1 (p15A replicon)	Amp^R	*tisB* expression under LexA-controlled promoter	(1)
Cia3	MC4100 *ara+*	-	-	Arabinose-tolerant strain	(2, 3)
Cia 30	MC4100 *ara+*	pBAD	Amp^R	Derived from pBAD-TOPO (invitrogen), *ara*BAD promoter, high-copy number (pBR322 replicon)	(3)
Cia27	MC4100 *ara+*	pBAD +1	Amp^R	pBAD expressing entire *tisB* gene	(3)
Cia28	MC4100 *ara+*	pBAD +42	Amp^R	pBAD expressing +42 *tisB* mRNA	(3)
AL43	MC4100 *ara+*	pBAD +1 G57C	Amp^R	pBAD expressing entire *tisB* gene, base substitution G57C	This study
AL44	MC4100 *ara+*	pBAD +42 G57C	Amp^R	pBAD expressing +42 *tisB* mRNA, base substitution G57C on pseudoknot stem 2 left arm	This study
AL46	MC4100 *ara+*	pBAD +42 C74G	Amp^R	pBAD expressing +42 *tisB* mRNA, base substitution C74G on pseudoknot stem 2 right arm	This study
AL47	MC4100 *ara+*	pBAD +42 G57C/C74G	Amp^R	pBAD expressing +42 *tisB* mRNA G57C/G74C compensatory mutant (Comp. 1)	This study
AL48	MC4100 *ara+*	pBAD +42 C74G/A75G	Amp^R	pBAD expressing +42 *tisB* mRNA, base substitution C74G/A75G on pseudoknot stem 2 right arm	This study
AL49	MC4100 *ara+*	pBAD +42 U56C/G57C	Amp^R	pBAD expressing +42 *tisB* mRNA, base substitution U56C/G57C on pseudoknot stem 2 left arm	This study
AL50	MC4100 *ara+*	pBAD +42 U56C/G57C C74G/A75G (Comp. 2)	Amp^R	pBAD expressing +42 *tisB* mRNA U56C/G57C C74G/A75G compensatory mutant (Comp. 2)	This study
Al51	MC4100 *ara+*	pBAD +42 G76C/C77G	Amp^R	pBAD expressing +42 *tisB* mRNA, base substitution G76C/C77G on pseudoknot stem 2 right arm	This study
AL52	MC4100 *ara+*	pBAD G54C/C55G	Amp^R	pBAD expressing +42 *tisB* mRNA, base substitution G54C/C55G on pseudoknot stem 2 left arm	This study
AL53	MC4100 *ara+*	pBAD +42 G54C/C55G G76C/C77G (Comp. 3)	Amp^R	pBAD expressing +42 *tisB* mRNA G54C/C55G G76C/C77G compensatory mutant (Comp. 3)	This study
AL54	MC4100 *ara+*	pBAD +42 C74G/C77G	Amp^R	pBAD expressing +42 *tisB* mRNA, base substitution C74G/C77G on pseudoknot stem 2 right arm	This study
AL55	MC4100 *ara+*	pBAD G54C/G57C	Amp^R	pBAD expressing +42 *tisB* mRNA, base substitution G54C/G57C on pseudoknot stem 2 left arm	This study
AL56	MC4100 *ara+*	pBAD +42 G54C/G57C C74G/C77G (Comp. 4)	Amp^R	pBAD expressing +42 *tisB* mRNA G54C/G57C C74G/C77G compensatory mutant (Comp. 4)	This study

(a) Nucleotides boxed in grey and black indicate mutations in the left or right arm of the RNA pseudoknot, respectively.

1. Vogel, J., Argaman, L., Wagner, E.G.H. and Altuvia, S. (2004) The small RNA IstR inhibits synthesis of an SOS-induced toxic peptide. *Curr. Biol.* **14**, 2271–2276.
2. Shimizu, H., Nishiyama, K. and Tokuda, H. (1997) Expression of *gpsA* encoding biosynthetic sn-glycerol 3-phosphate dehydrogenase suppresses both the LB− phenotype of a *secB* null mutant and the cold-sensitive phenotype of a *secG* null mutant. *Mol. Microbiol.* **26**, 1013–1021.
3. Unoson, C. and Wagner, E.G.H. (2008) A small SOS-induced toxin is targeted against the inner membrane in *Escherichia coli*. *Mol. Microbiol.* **70**, 258–270.

Table S2: Primers used in this study

Name	Sequence (5' to 3')(a, b)	Template (d)	Used for (c, d)
T7.HH.tis42 (1)	GAAATTAATACGACTCACTATAGGGAGACTTAGTG CTGATGAGTCCGTGAGGACGAAACGGTACCCGGT ACCGTCCACTAAGTCCTGGCTGAAACGG**	pJV 974-1 (2) or pJV974_C74G (this study)	PCR template for T7 transcription of HH(**e**) +42 *tisB* mRNAs
3' tisAB reverse (1)	AAAAGGGGAGCGGTTTCCCG	pJV 974-1 (2)	PCR template for T7 transcription of *tisB* mRNAs
T7.HH.tisB106(1)	GAAATTAATACGACTCACTATAGGGAG**TTGACATA** CTGATGAGTCCGTGAGGACGAAACGGTACCCGGT ACCGTCTATGTCAACAAGCACAACGTTTCTCC	pJV 974-1 (2)	PCR template for T7 transcription of HH +106 *tisB* mRNAs
splint.tisB.5.prime (1)	GTGCTTGTTGACATAACACAGTGTGCTCAC	Partial complementarity to 5' FAM-105 and +106 *tisB* mRNA	Ligation 5'adapter with FAM 42-105 to +106 tisB mRNA
5'FAM 42-105 (1)	FAM-CACUAAGUCCUGGCUGAAACGGGUGGUGCCGUC AGCGCCUUAACCCCGCGUGAGCAC CUGUGU	Partial complementarity to splint.tisB.5.prime	5'adapter RNA, splint ligation
tisb rev 1 (1)	CCAGCTGACGTACCTTTC	+42 or G57C *tisB* mRNA	Reverse transcription, structural probing
toe tisB (1)	TCAGGTATTTCAGAACAGCA	*tisB* mRNA variants	Toeprint
T7HH_G57C_for	GAAATTAATACGACTCACTATAGGGAG**ACTTAGTG** CTGATGAGTCCGTGAGGACGAAACGGTACCCGGT ACCGTC**CACTAAG**TCCTGGCT**C**AAACGGGTGGT** **	pJV 974-1 (2) or pJV974_C74G (This study)	PCR template for T7 transcription of HH* +42 *tisB* G57C mRNAs
T7HH_G54A_for	GAAATTAATACGACTCACTATAGGGAG**ACTTAGTG** CTGATGAGTCCGTGAGGACGAAACGGTACCCGGT ACCGTC**CACTAAG**TCCTG**A**CTGAAACGGGTGGT	pJV 974-1 (2)	PCR template for T7 transcription of HH* +42 G54A *tisB* mRNAs
T7HH_C55A_for	GAAATTAATACGACTCACTATAGGGAG**ACTTAGTG** CTGATGAGTCCGTGAGGACGAAACGGTACCCGGT ACCGTC**CACTAAG**TCCTGG**A**TGAAACGGGTGGT	pJV 974-1 (2)	PCR template for T7 transcription of HH* +42 C55A *tisB* mRNAs
G57C_for	AGTCCTGGCTCAAACGGGTGG	pBAD_+1 or pBAD +42 (3)	*Dpn*I mutagenesis to create pBAD_+1_G57C or pBAD +42_G57C
+42_G57C_rev	TAGTCTGGAGAAACAGTAGAGAGTTG	pJV 974-1 or pBAD_+1 or pBAD +42 (3)	*Dpn*I mutagenesis to create pBAD_+1_G57C or pBAD +42_G57C
+1_G57C_rev	TAGTGCGCCGGGTAACGA	pJV 974-1 (2) or pBAD_+1 (3) or pBAD +42 (3)	*Dpn*I mutagenesis to create pBAD_+1_G57C or pBAD +42_G57C
C74G_for	GGTGCCGTCACGGCCTTAACCCCGC	pJV 974-1 or pBAD +42 (3) or pBAD +42_G57C (This study)	*Dpn*I mutagenesis to create pJV 974-1_C74G or pBAD +42_C74G or pBAD +42_G57C/C74G (Comp. 1)
C74G_rev	ACCCGTTTCAGCCAGGAC	pJV 974-1 or pBAD +42 (3) or pBAD +42_G57C (This study)	*Dpn*I mutagenesis to to create pJV 974-1_C74G or pBAD +42_C74G or pBAD +42_G57C/C74G (Comp. 1)
42 C74G/A75G for	GTGGTGCCGTggGCGCCTTAAC	pBAD +42 or pBAD +42 U56C/G57C	*Dpn*I mutagenesis to create pBAD +42 C74G/A75G or pBAD +42 U56C/G57C C74G/A75G (Comp. 2)
42 C74G/A75G rev	CCGTTTCAGCCAGGACTTA	pBAD +42 or pBAD +42 U56C/G57C	*Dpn*I mutagenesis to create pBAD +42 C74G/A75G or pBAD +42 U56C/G57C C74G/A75G (Comp. 2)
42 G76C/C77G for	GGTGCCGTCAcgGCCTTAACCCCGC	pBAD +42 or pBAD G54C/C55G	*Dpn*I mutagenesis to create pBAD +42 G76C/C77G or pBAD +42 G54C/C55G G76C/C77G (Comp. 3)
42 G76C/C77G rev	ACCCGTTTCAGCCAGGAC	pBAD +42 or pBAD G54C/C55G	*Dpn*I mutagenesis to create pBAD +42 G76C/C77G or pBAD +42 G54C/C55G G76C/C77G (Comp. 3)
42 C74G/C77G for	GTGGTGCCGTgaggGCCTTAACCC	pBAD +42 or pBAD G54C/G57C	*Dpn*I mutagenesis to create pBAD +42 C74G/C77G or pBAD +42 G54C/G57C C74G/C77G (Comp. 4)
42 C74G/C77G rev	CCGTTTCAGCCAGGACTTAGTG	pBAD +42 or pBAD G54C/G57C	*Dpn*I mutagenesis to create pBAD +42 C74G/C77G or pBAD +42 G54C/G57C C74G/C77G (Comp. 4)
42 U56C/G57C for	AAGTCCTGGCccAAACGGGTGG	pBAD +42 or p BAD C74G/A75G	*Dpn*I mutagenesis to create pBAD +42 U56C/G57C or pBAD +42 U56C/G57C C74G/A75G (Comp. 2)
42 U56C/G57C rev	AGTCTGGAGAAACAGTAGAG	pBAD +42 or pBAD C74G A75G	*Dpn*I mutagenesis to create pBAD +42 U56C G57C or

			pBAD +42 U56C G57C / C74G A75G (Comp. 2)
42 G54C/C55G for	CTAAGTCCTGcgTGAAACGGGTGG	pBAD +42 or pBAD G76C/C77G	*Dpn*I mutagenesis to create pBAD +42 G54C/C55G or pBAD +42 G54C/C55G G76C/C77G (Comp. 3)
42 G54C/C55G rev	TCTGGAGAAACAGTAGAG	pBAD +42 pBAD G76C/C77G	*Dpn*I mutagenesis to create pBAD +42 G54C/C55G or pBAD +42 G54C/C55G G76C/C77G (Comp. 3)
42 G54C/G57C for	CTAAGTCCTGcctcAAACGGGTGG	pBAD +42 or pBAD C74G C77G	*Dpn*I mutagenesis to create pBAD +42 G54C/G57C or pBAD +42 G54C/G57C C74G/C77G (Comp. 4)

(a) Red and green – complementary sequences that form the P1 stem of the Hammerhead
(b) **N**: Point mutation in the corresponding PCR product
(c) Nucleotides boxed in grey and black indicate mutations in the left or right arm of the pseudoknot, respectively.
(d) See Table S1 for plasmid information
(e) HH: Hammerhead ribozyme-containing RNA.

1. Romilly, C., Deindl, S. and Wagner, E.G.H. (2019) The ribosomal protein S1-dependent standby site in *tisB* mRNA consists of a single-stranded region and a 5' structure element. *Proc. Natl. Acad. Sci. U.S.A.* **116**, 15901–15906.
2. Vogel, J., Argaman, L., Wagner, E.G.H. and Altuvia, S. (2004) The small RNA IstR inhibits synthesis of an SOS-induced toxic peptide. *Curr. Biol.* **14**, 2271–2276.
3. Unoson, C. and Wagner, E.G.H. (2008) A small SOS-induced toxin is targeted against the inner membrane in *Escherichia coli*. *Mol. Microbiol.* **70**, 258–270.

Table S3: Pseudoknot structure predictions for type I TA toxin mRNAs

TA system	Sequence input (50 nts upstream sRNA anti-toxin base-pairing site) **(c)**	Pseudo / total hits (ΔG < –6 kcal/mol) **(a,b)**
tisB (1)	5'-CACTAAGTCCTGGCTGAAACGGGTGGTGCCGT CAGCGCCTTAACC-3'	13 / 19
	CACTAAGTCCTGGCTGAAACGGGTGGTGCCGTCAGCGCCTTAACC<br>5'- (((((...(((([[[[[..)))))))))...]]]]]......... -3'<br>(-9.53 kcal/mol)*	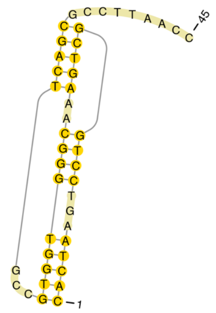
	CACTAAGTCCTGGCTGAAACGGGTGGTGCCGTCAGCGCCTTAACC<br>5'- .....((([[[[))))....]]]].(((((.....)))))...... -3'<br>(-8.31 kcal/mol)	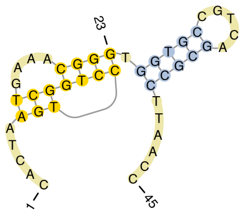
	CACTAAGTCCTGGCTGAAACGGGTGGTGCCGTCAGCGCCTTAACC<br>5'- (((((......[[[....[[[[.)))))]]]]....]]]...... -3'<br>(-8.20 kcal/mol)	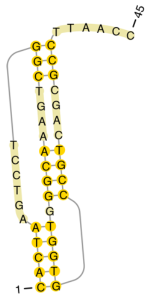
zor (2)	TTTTAAGTCCTGGCTGCCGGACGGGTGGTGCCGCAG GCGGTGCCC	9 / 16
	TTTTAAGTCCTGGCTGCCGGACGGGTGGTGCCGCAGGCGGTGCCC<br>5'- ......((((.........))))....((((((((....)))))))). -3′<br>(-11.44 kcal/mol)*	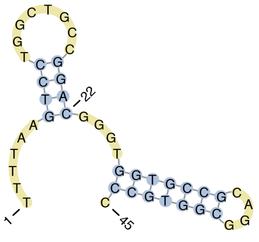
	TTTTAAGTCCTGGCTGCCGGACGGGTGGTGCCGCAGGCGGTGCCC<br>5'- ......((((..[[[[[[)))).]]]]]]((((....))))..... -3′<br>(-10.25 kcal/mol)	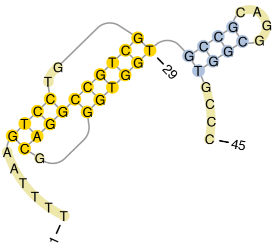

	TTTTAAGTCCTGGCTGCCGGACGGGTGGTGCCGCAGGCGGTGCCC<br>5'- ......((((...[[[[.))))(((((.....]]]]....))))) -3'<br>(-9.19 kcal/mol)	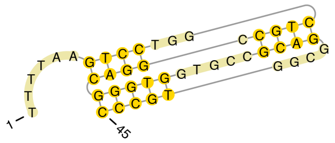
shoB (3)	CCCCCAUUGAAACGAGUGGUGUCGUCAAAGCUCUG GUGUGGAGUG	2 / 2
	CCCCCAUUGAAACGAGUGGUGUCGUCAAAGCUCUGGUGUGGAGUG<br>5'- ....(((((..[[[[.)))))]]]]....((((.......)))). -3'<br>(-6.97 kcal/mol)*	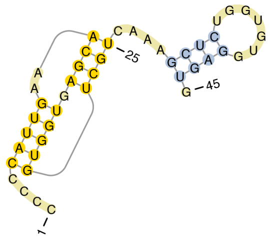

(a) Most stable structures predicted by the HotKnots software (Dirks & Pierce model, set of parameters DP09, 37°C) (http://www.rnasoft.ca/cgi-bin/RNAsoft/HotKnots/hotknots.pl) (4)
(b) 2D structures obtained with PseudoViewer software (http://wilab.inha.ac.kr/pseudoviewer/) (5, 6)
(c) Nucleotide interactions to form RNA helices are indicated by ( ) (stem 1) or [ ] (stem 2).

Information on Table S3:

Three type I TA systems from *E. coli* were considered: *tisB-istR1*, *zor-orz,* and *shoB-ohsC*. A region encompassing 50 nt directly upstream of the anti-toxin sRNA binding sites in the mRNAs was used as input for HotKnot software. The Table displays the results using the Dirks & Pierce model, set of parameters DP09, 37°C, with a cut-off set up with a ΔG below –6 kcal/mol. For each toxin mRNA, the number of pseudoknot hits over the total number of structures predicted is indicated. Representative examples of pseudoknot variants and their corresponding ΔG-values, as well as secondary structures obtained with Pseudoviewer (v3) software, are shown.

1. Vogel, J., Argaman, L., Wagner, E.G.H. and Altuvia, S. (2004) The small RNA IstR inhibits synthesis of an SOS-induced toxic peptide. *Curr. Biol.* **14**, 2271–2276.
2. Wen, J., Won, D. and Fozo, E.M. (2014) The ZorO-OrzO type I toxin–antitoxin locus: repression by the OrzO antitoxin. *Nucleic Acids Res.* **42** 1930–1946.
3. Fozo, E.M., Kawano, M., Fontaine, F., Kaya, Y., Mendieta, K.S., Jones, K.L., Ocampo, A., Rudd, K.E. and Storz, G. (2008) Repression of small toxic protein synthesis by the Sib and OhsC small RNAs. *Mol. Microbiol.* **70** 1076–1093.
4. Andronescu, M.S., Pop, C. and Condon, A.E. (2010) Improved free energy parameters for RNA pseudoknotted secondary structure prediction. *RNA* **16**, 26–42.
5. Han, K., Lee, Y. and Kim, W. (2002) PseudoViewer: automatic visualization of RNA pseudoknots. *Bioinformatics* **18**, S321–S328.
6. Byun, Y. and Han, K. (2009) PseudoViewer3: generating planar drawings of large-scale RNA structures with pseudoknots. *Bioinformatics* **25**, 1435–1437.

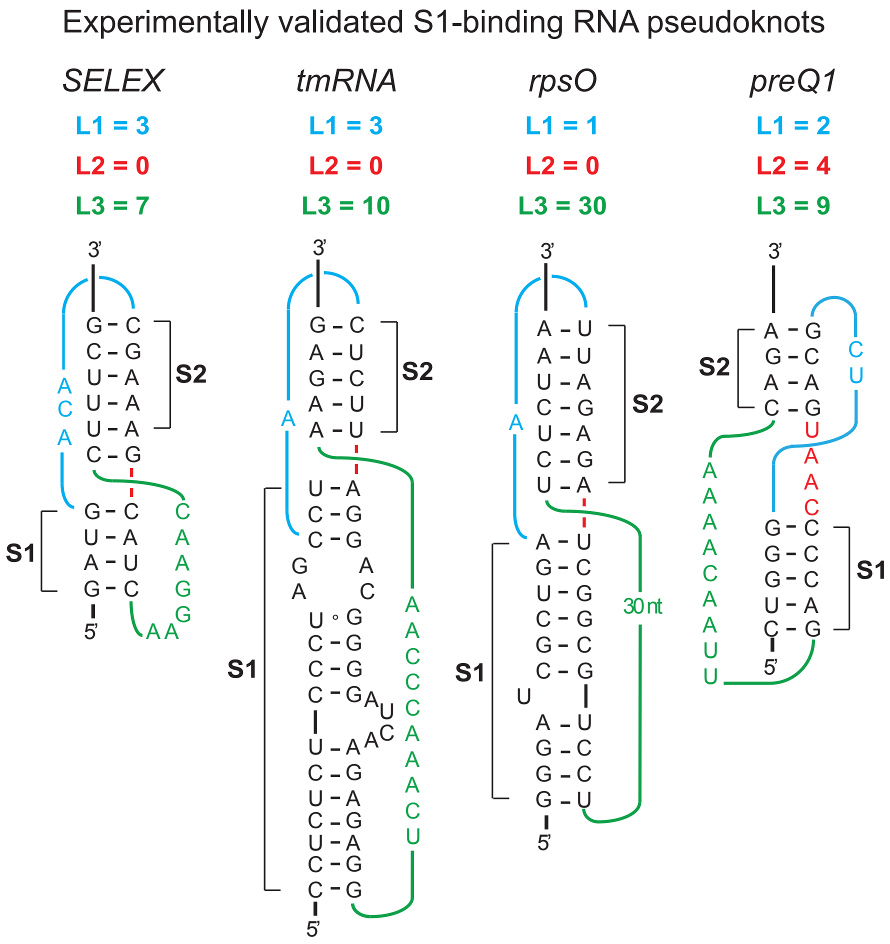

Figure S1. **Secondary structures of known S1-bound RNA pseudoknots.**

SELEX-based (1), tmRNA (2), rpsO (3), and preQ1 (4)

1. Ringquist, S., Jones, T., Snyder, E.E., Gibson, T., Boni, I. and Gold, L. (1995) High-affinity RNA ligands to *Escherichia coli* ribosomes and ribosomal protein S1: Comparison of natural and unnatural binding sites. *Biochemistry* **34**, 3640–3648.
2. Bordeau, V. and Felden, B. (2002) Ribosomal protein S1 induces a conformational change of tmRNA; more than one protein S1 per molecule of tmRNA. *Biochimie* **84**, 723–729.
3. Duval, M., Korepanov, A., Fuchsbauer, O., Fechter, P., Haller, A., Fabbretti, A., Choulier, L., Micura, R., Klaholz, B.P., Romby, P., et al. (2013) *Escherichia coli* ribosomal protein S1 unfolds structured mRNAs onto the ribosome for active translation initiation. *PLoS Biol*. **11**, e1001731.
4. Lund, P.E., Chatterjee, S., Daher, M. and Walter, N.G. (2020) Protein unties the pseudoknot: S1-mediated unfolding of RNA higher order structure. *Nucleic Acids Res*. **48**, 2107–2125.

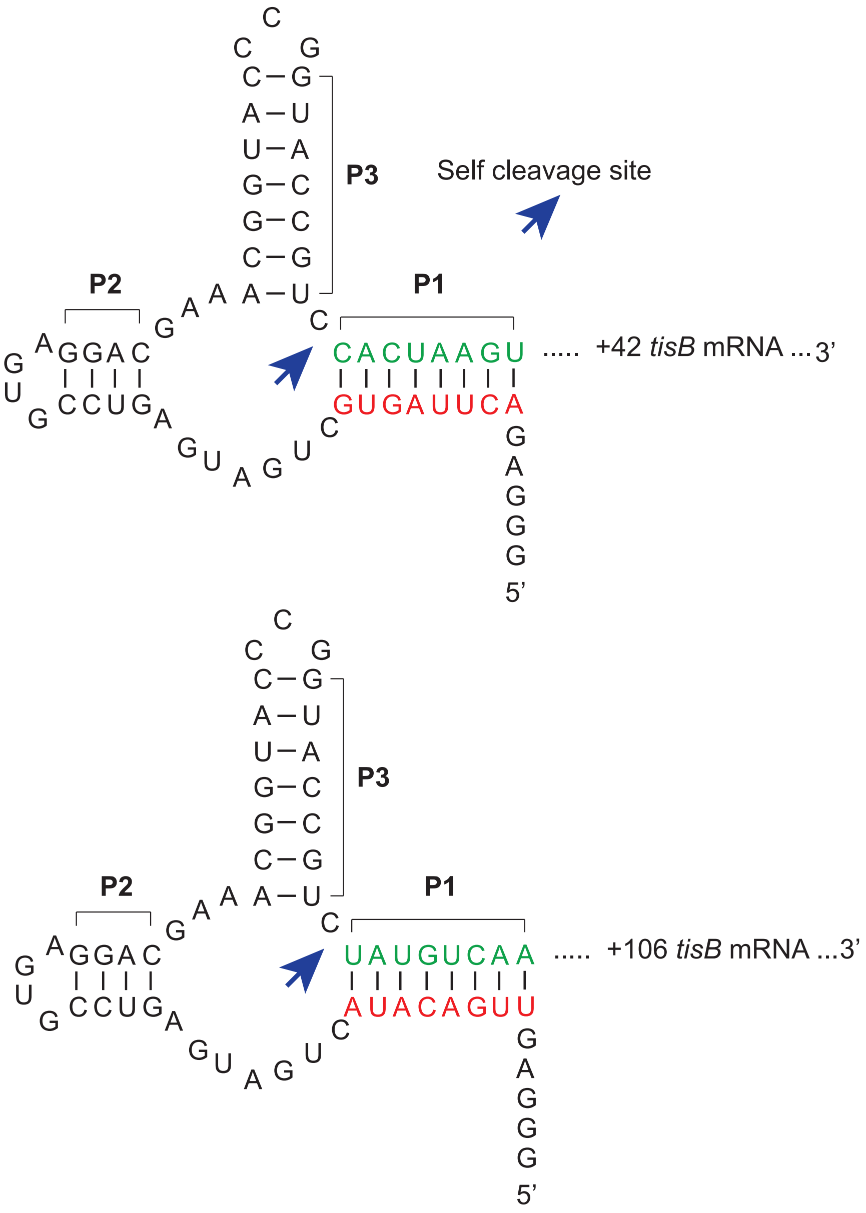

Figure S2. **Hammerhead template for specific cleavage of the +42 *tisB* mRNA sequence.** The P1, P2, and P3 secondary structures guide the specific cleavage of the pre-mRNA at the position indicated by the blue arrow. The nucleotides in green are the 5'end terminal nucleotides of the +42 or +106 tisB mRNA; complementary nt's in red. The same color code is used in the primer Table S2.

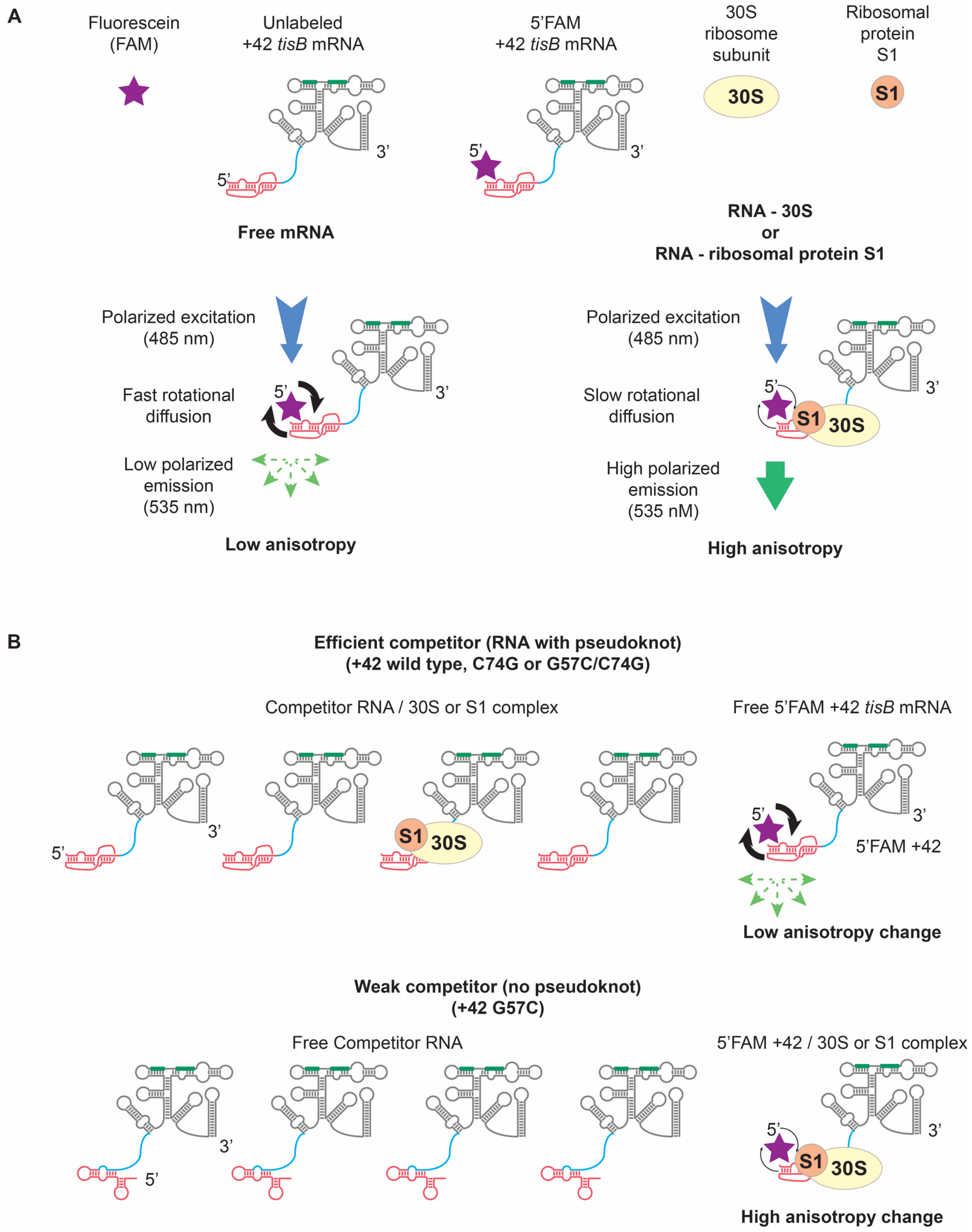

Figure S3. **Schematic representation of anisotropy change measurements upon 30S subunit or S1 r-protein binding, with or without competitor unlabeled mRNA.**

For more information consult (1) and Materials and Methods.

1. Romilly, C., Deindl, S. and Wagner, E.G.H. (2019) The ribosomal protein S1-dependent standby site in *tisB* mRNA consists of a single-stranded region and a 5' structure element. *Proc. Natl. Acad. Sci. U.S.A.* **116**, 15901–15906.

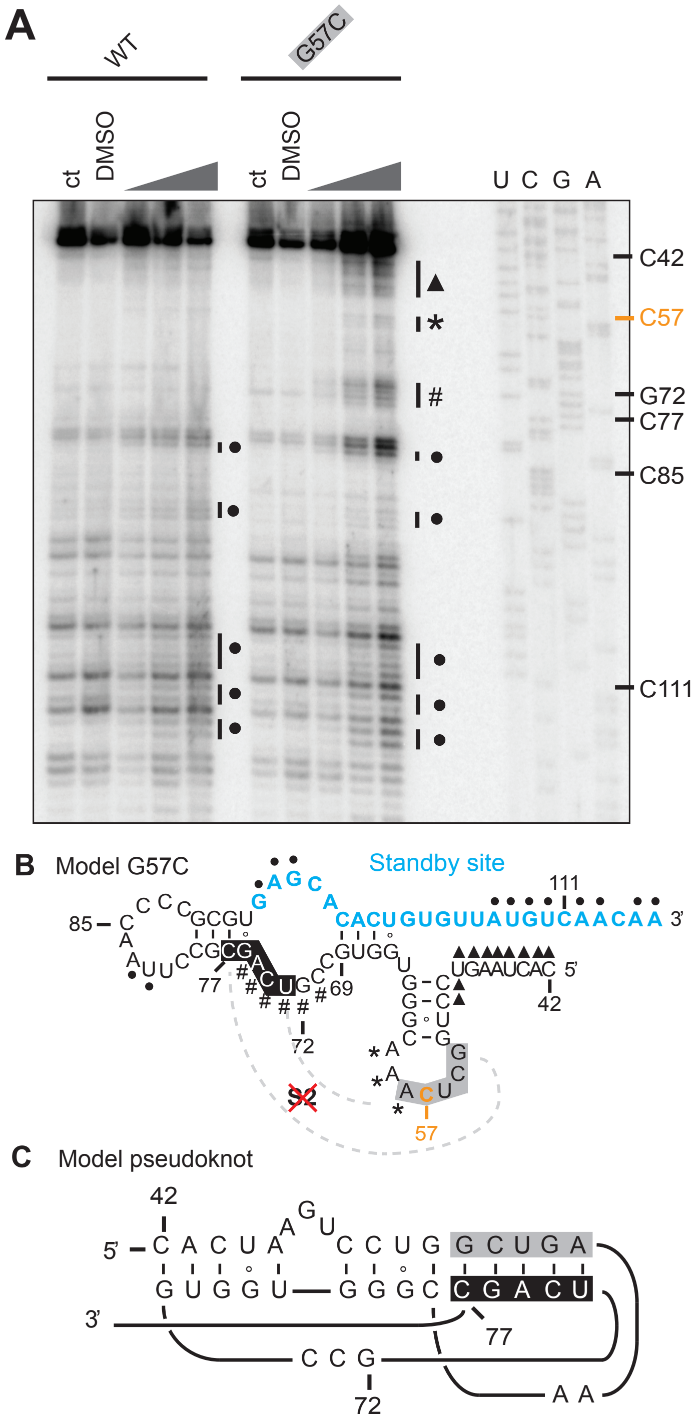

Figure S4. **SHAPE structural probing of wild-type and G57C *tisB* mRNA.**

(A) SHAPE structural probing was carried out on wild-type +42 *tisB* mRNA or its G57C mutant counterpart. 2'-O-adduct signals were obtained by primer extension (Materials and Methods).

(B) Changes in SHAPE reactivity are highlighted on the *tisB* mRNA secondary structure. Black dots: induced SHAPE reactivity shared between both mRNAs tested. Increased SHAPE reactivities in the G57C mRNA compared to WT are highlighted with, #, *, and triangles. Less structural propensity likely prevents formation of helix S2 as indicated (crossed-out S2).

(C) Redrawn model of the proposed pseudoknot for comparison, in which relative changes can be assessed.

More information of the SHAPE method.

To address structural differences, SHAPE (selective 2'-hydroxyl acylation analyzed by primer extension) structural probing was used to monitor differences in RNA backbone flexibility between the wild-type and G57C mutant RNAs. The SHAPE reagent probes the reactivity of the 2'-OH group in RNA and correlates with local nucleotide flexibility. A pseudoknot-like structure in the 5'UTR of +42 *tisB* mRNA should exhibit a lower degree of flexibility compared to that of the G57C mutant mRNA in which the pseudoknot is disrupted. Both mRNAs were treated with increasing concentrations of 1-methyl-7-nitroisatoic anhydride (1M7; half-life at 37°C: 17 sec) diluted in DMSO (1). The 2'-OH-adducts trigger reverse transcription pauses at the sites of modification. Control reactions in water and in presence of DMSO indicated no RT-dependent pauses (see above). For both mRNAs, SHAPE reactivity patterns were detected within the standby site region (pos. 93 to 116), previously shown to be single-stranded by chemical and enzymatic probing (2-3). Several additional SHAPE-induced RT pauses were observed at positions upstream of the standby site, but only in the G57C mutant mRNA. Most strikingly, the proposed loop-loop interacting sequences, normally involved in pseudoknot formation, were more flexible in the mutant mRNA compared to wild-type +42. In the G57C mutant, the A triplet at position 58-60 (marked with asterisks *) and the region 71-75 (marked with hash tags #) exhibited enhanced pause signals upon increasing concentrations of 1M7. Finally, the 5'-most nucleotides of the *tisB* mRNA also displayed enhanced reactivity to the 1M7 SHAPE reagent compared to wild-type mRNA, tentatively supporting the pseudoknot prediction in Figure 1D and 4A in the main paper.

1. Smola, M.J., Rice, G.M., Busan, S., Siegfried, N.A. and Weeks, K.M. (2015) Selective 2′-hydroxyl acylation analyzed by primer extension and mutational profiling (SHAPE-MaP) for direct, versatile, and accurate RNA structure analysis. *Nat. Protoc*. **10**, 1643–1669.
2. Darfeuille, F., Unoson, C., Vogel, J. and Wagner, E.G.H. (2007) An antisense RNA inhibits translation by competing with standby ribosomes. *Mol. Cell*. **26**, 381–392.
3. Romilly, C., Deindl, S. and Wagner, E.G.H. (2019) The ribosomal protein S1-dependent standby site in *tisB* mRNA consists of a single-stranded region and a 5' structure element. *Proc. Natl. Acad. Sci. U.S.A.* **116**, 15901–15906.

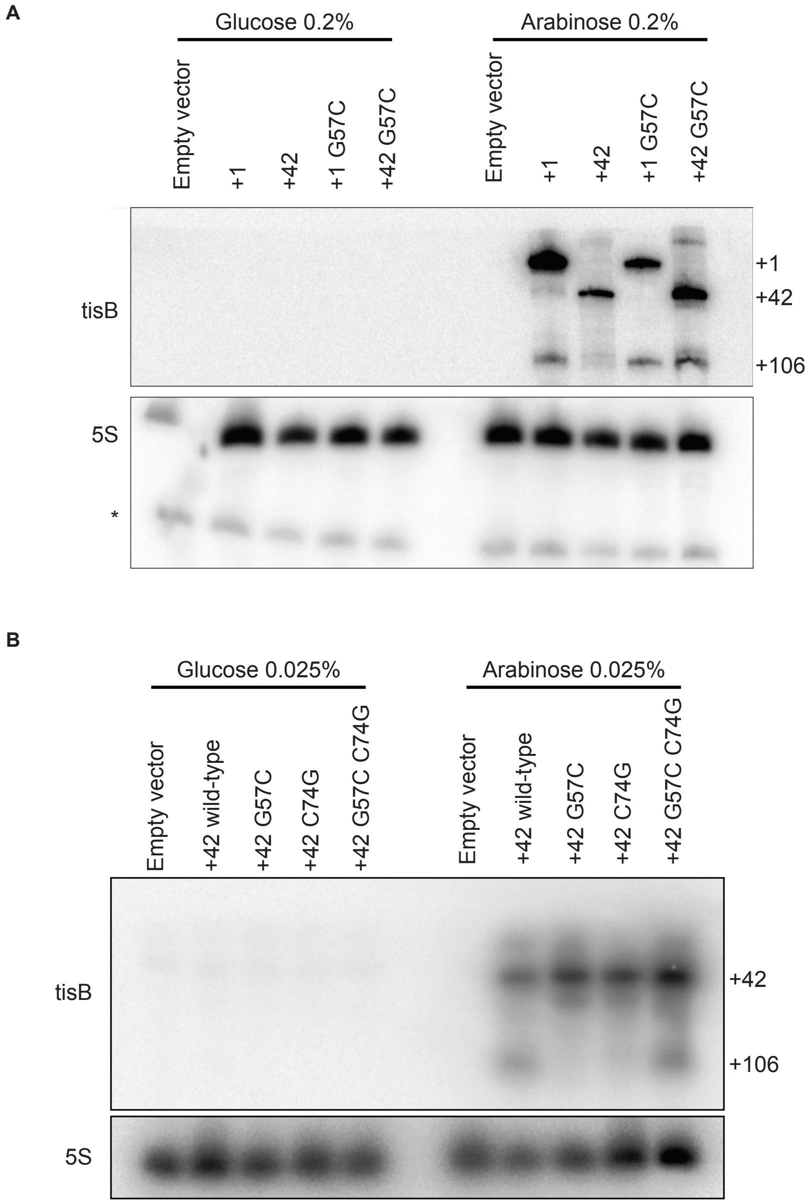

Figure S5. **Northern blot analysis on strains expressing *tisB* mRNAs.** (A) Northern blot analysis corresponding to the toxicity assay in Figure 3. (B) Same analysis, referring to toxicity assay in Figure 5. In both cases, primer 267 (base-pairing between position 160 and 180) was used to simultaneously detect the +1, +42 and +106 *tisB* mRNAs. A 5S probe was used as a loading control. Total RNA was extracted 20 min post induction with glucose or arabinose, respectively.

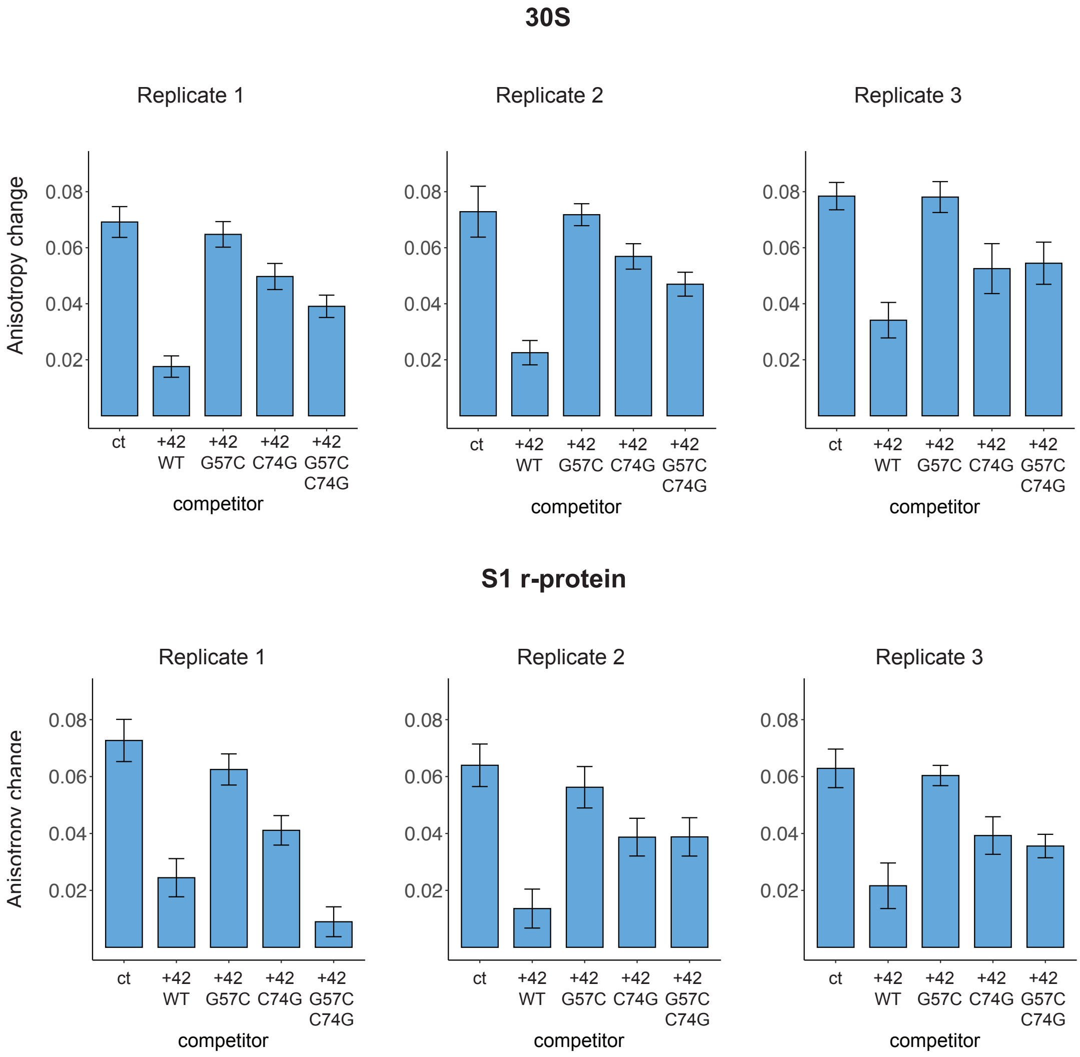

Figure S6. **Binding assay triplicate break-up (triplicate)**. The data set shows the individual runs used to give the values in Figure 4C in the main paper.
